# Supplementary material for: Adoption of Digital Vaccination Services: It Is the Click Flow, Not the Value—An Empirical Analysis of the Vaccination Management of the COVID-19 Pandemic in Germany
Source: Vaccines (Basel). 2023 Mar 28;11(4):750. doi: 10.3390/vaccines11040750 (PMC10145467; doi:10.3390/vaccines11040750)
Supplement: Supplementary file 1 [file vaccines-11-00750-s001.zip › vaccines-2291699-supplementary.pdf]

lfdn	dispcode	lastpage	duration	Federal	Vaccinat.	Role	Health	Age	Gender	Education	Vaccin.	Priorisati	Type of	Vacc.	Healt	Work	Campaign	Personal_	Personal_	Personal_	Commun	Commun	Commun	Data_Res	Data_Res	Data_Res	Integ_Pro	Integ_Pro	Integ_Pro	Satis_1	Satis_2	Satis_3	Satis_4	Intent_Ad	Intent_Ad	Consum_	Consum_	Consum_	Usability_	Usability_	Perc_Valu	Perc_Valu	Sec_Risk_	Sec_Risk_	Image_ba	Image_ba	Perc_Dep	Perc_Dep	Techn_An	Techn_An	Ideol_Bar	Ideol_Bar	Trad_Barr	Trad_Barr	Ind_Inerti	Ind_Inerti
1	31	5941582	253			1	2		1	6	1	6	3					5	3	3	6	6	6	7	7	7	4	4	4	6	7	7	5	7	7	1	7	1	1	1	1	7	4	4	1	1	1	1	1	1	1	1	4	2	2	1
2	31	5941582	1483			1	1		5	2	2	5	2		1	5	4	1	1	1	1	1	1				1	1	1	1	1	1	1	7	1	1	1	1	7	7	7	7	1	1	7	7	1	1	1	1	1	2	4	6	3	3
3	31	5941582	1515			1	1		5	1	1	1	1		1	8	8	1	1	1	1	1	1	1	1	1	1	1	1	1	1	1	1	1	1	1	1	1	7	7	7	7	1	1	7	7	1	1	1	1	1	1	1	1	1	1
4	31	5941582	695			1	2		2	2	1	1	3					7	7	7	7	7	7	7	7	7	7	7	7		1	1	1	1	1	1	1	1	7	7	7	7	1	1	7	7	1	1	1	1	1	1	1	1	1	1
5	31	5941582	1458			3	1		1	6	1	6	4		1	16	5	4	4	4	4	4	4	4	4	4	4	4	4	4	4	4	4	4	4	4	4	4	4	4	4	4	4	4	4	4	4	4	4	4	4	4	4	4	4	4
6	31	5941582	54			1	1		1	6	1	6	1		1	3	6	4	4	4	4	4	4	4	4	4	4	4	4	4	4	4	4	4	4	4	4	4	4	4	4	4	4	4	4	4	4	4	4	4	4	4	4	4	4	4
7	31	5941582	214			1	1		5	3	2	4	1		1	78	12	6	5	3	5	4	4	4	4	4	6	4	5	5	5	5	5	5	5	5	5	5	3	3	3	3	5	5	2	2	4	4	4	4	4	4	5	5	5	5
8	31	5941582	246	12	1	1	2	5	5	6	1	4	3	1				7	7	7	7	7	7	5	4	4	4	5	5	7	7	7	7	7	7	1	1	1	1	1	1	7	1	1	1	1	1	1	1	1	1	1	5	2	1	1
9	31	5941582	358	12	1	1	2	2	1	5	1	6	6	1				7	5	4	7	7	4	5	5	5	5	7	3	3	3	7	5	4	7	2	1	1	1	3	5	7	1	1	1	1	1	1	1	1	2	1	4	3	2	4
1	31	5941582	458	12	1	1	2	5	5	6	1	6	1	1				7	6	6	7	7	7	7	7	7	7	4	4	7	7	7	7	7	7	1	1	1	1	1	1	7	1	1	1	1	1	1	1	1	1	1	1	1	1	1
11	31	5941582	623	12	3	1	2	5	5	3	1	5	1	1				5	5	4	4	4	4	4	4	4	4	3	4	4	4	4	4	3	3	2	2	2	3	3	4	5	4	3	4	5	4	3	2	2	4	4	6	5	2	3
12	31	5941582	351	12	1	1	2	3	5	3	1	4	3	1				7	5	6	7	4	4		5	5	4	4	4	7	7	4	5	4	7	1	1	1	1	1	1	7	4	4	1	1	1	1	1	1	1	1	4	4	1	1
13	31	5941582	752	12	1	1	2	5	5	6	1	5	3	1				7	7	7	7	7	7	5	5	5	6	5	6	7	5	5	5	5	3	1	1	1	1	1	1	7	4	4	5	6	2	3	2	2	3	3	6	5	3	5
14	31	5941582	37	12	1	1	2	3	5	6	1	6	6	1				6	5	5	4	6	5	2	2	2	5	6	6	6	6	6	6	4	4	2	5	2	1	4	2	6	3	3	3	3	2	2	2	2	2	2	3	3	2	5
15	31	5941582	47	12	1	1	1	4	5	4	1	6	1	1	3	26	7	7	7	7	7	7	7	6	6	6	7	7	6	7	7	7	7	3	7	1	1	1	1	1	1	7	1	1	1	3	1	1	1	1	1	1	3	2	2	4
16	31	5941582	412	12	1	1	2	7	5	6	1	2	3	1				7	7	7	7	7	7	5	6	6	4	6	4	7	7	7	7	3	6	1	1	1	1	1	2	6	2	2	1	1	1	1	1	1	1	1	4	3	1	4
17	31	5941582	366	12	1	1	2	2	1	5	1	6	3	1				5	4	4	6	6	4	6	6	7	4	4	3	7	7	7	7	5	6	1	1	1	1	2	1	7	1	1	2	2	1	1	1	1	1	1	4	2	3	4
18	31	5941582	329	12	1	1	2	8	1	4	2	1	1	1				7												7	7	7	7	7	7	1	1	1	1	1	1	7	4	4	7	7	1	1	3	2	5	7	7	4	1	7
19	31	5941582	474	12	1	1	2	3	2	5	1	5	1	2				2	2	3	5	2	1	1	1	1	2	6	2	5	2	1	1	2	1	7	7	4	3	6	6	2	4	4	2	3	7	7	3	2	5	7	7	4	3	5
2	31	5941582	246	12	1	1	2	6	5	6	1	7	1	2				1	1	1	1	1	1	2	2	1	1	1	2	1	1	5	1	1	1	1	1	2	7	7	7	7	4	4	1	1	1	1	1	1	1	1	1	1	1	1
21	31	5941582	354	12	1	1	2	5	5	6	1	4	3	1				3	2	2	5	5	6	1	2	2	6	6	5	4	4	5	5	2	2	5	5	5	3	7	2	5	4	4	6	5	2	2	2	2	4	4	6	6	1	3
22	31	5941582	378	12	3	1	2	3	1	4	6	7	3	1				7	7	7	7	7	7	7	7	7	7	7	7	7	7	7	7	7	7	1	1	1	1	1	1	7	4	4	1	1	1	1	1	1	1	1	1	4	1	5
23	31	5941582	492	12	4	1	2	6	5	6	1	3	2	1				5	3	3	5	4	3	3	3	3	5	5	5	4	4	5	5	4	4	2	2	2	2	3	2	6	2	2	3	3	2	2	2	2	2	2	6	5	2	2
24	31	5941582	252	12	1	1	2	4	1	3	1	5	1	1				7	6	6	7	7	4	2	3	2	3	3	3	7	7	7	7	7	7	1	1	1	1	1	1	7	3	3	1	1	1	1	1	1	1	1	6	6	1	7
25	31	5941582	41	12	1	1	2	4	5	4	1	5	3	1				6	2	2	4	4	4	2	2	2	5	3	4	2	2	5	4	6	6	4	2	2	2	4	6	6	4	4	1	1	1	1	1	1	1	1	4	4	2	2
26	31	5941582	381	12	1	1	2	5	5	6	1	4	3	1				7	5	6	6	6	6	6	6	6	7	6	6	6	6	6	6	6	6	1	2	1	2	2	2	7	2	2	3	3	5	3	2	1	3	2	5	4	3	5

27	31	5941582	321	11	3	1	2	3	5	6	1	6	3	1				2	2	2	1	4	4	3	2	1	3	2	4	3	1	1	2	2	2	6	2	2	2	2	2	2	3	3	1	1	2	2	2	1	1	2	3	2	1	2
28	31	5941582	476	12	1	1	2		5	5	1	6	5	1				7	5	4	6	7	5	4	4	4	5	5	4	5	5	6	5	3	6	3	3	3	1	2	2	5	4	4	2	2	2	2	2	2	2	2	4	4	3	5
29	31	5941582	48	12	1	1	1	4	5	4	1	6	1	1	1	2	3	6	4	5	7	7	4	2	2	2	4	5	4	6	5	7	7	7	7	5	5	5	2	7	6	3	6	6	3	3	5	6	4	5	6	5	5	5	3	3
3	31	5941582	34	12	1	1	2	4	5	4	1	2	1	1				6	6	6	6	5	5	7	7	7	5	5	4	6	6	7	7	5	7	3	3	1	1	2	2	6	3	2	1	1	1	1	1	1	1	1	2	2	2	4
31	31	5941582	393	12	1	1	2	4	5	3	1	5	5	1				7	6	7	7	6	5	6	6	6	7	7	7	7	7	7	7	7	7	1	2	1	1	1	1	7	2	2	1	1	1	1	1	1	1	1	6	6	2	4
32	31	5941582	412	12	1	1	2	4	5	4	1	6	3	1				7	7	7	7	7	6	4	6	6	5	7	6	7	7	7	7	5	7	1	1	1	1	1	1	7	2	3	1	1	1	1	1	1	1	1	7	4	2	7
33	31	5941582	646	12	1	1	2	6	5	3	2	6	1	1				6	5		7	7		5	5	5		6	7	5	6	7	6	7	7	6	1	1	3	3	2	7	2	2	2	2	2	2	1	1	2	2	3	3	1	4
34	31	5941582	499	12	1	1	2	5	1	6	1	4	3	1				6	5	6	7	7	7	6	6	6	7	7	7	6	6	6	5	7	7	1	1	1	3	2	2	2	4	4	5	4	5	2	2	2	2	2	2	2	2	1
35	31	5941582	573	12	1	1	2	6	5	3	1	6	1	1				6	6	6	7	6	6	6	6	6	6	5	6	6	6	6	5	5	6	2	1	1	1	2	2	7	5	2	2	2	3	3	1	4	3	3	6	5	3	4
36	31	5941582	896	12	1	1	2	3	1	4	1	6	1	1				7	5	5	7	7	7	5	5	5	6	6	6	6	6	6	6	6	6	2	1	1	2	3	2	7	2	2	2	2	1	1	1	1	1	1	4	4	2	3
37	31	5941582	777	12	1	1	2	6	1	6	1	4	3	2				6	4	6	7	5	7	6	7	6	6	7	6	7	6	7	7	6	7	2	1	1	2	1	2	7	3	3	2	1	2	3	1	2	3	2	4	1	2	4
38	31	5941582	781	12	1	1	2	6	5	4	1	6	3	1				7	6	6	6	7	5	7	6	6	6	6	6	6	6	7	7	5	6	2	2	2	1	1	1	7	2	2	6	7	6	4	5	4	7	4	7	7	1	6
39	31	5941582	623	12	1	1	2	4	5	3	1	6	3	1				7	4	4	7	7	7	6	6	6	4	6	5	7	7	7	7	7	7	1	1	1	1	2	1	7	6	6	3	3	2	1	1	2	2	2	3	3	2	6
4	31	5941582	523	12	1	1	2	7	1	6	2	2	3	1				3	1	1	1	1	1	1	3	1	1	1	1	1	1	1	1	4	1	7	3	7	4	7	5	4	3	4	7	5	7	6	7	5	7	6	7	7	4	3
41	31	5941582	823	12	1	1	2	3	5	6	1	7	5	1				3	4	3	7	6	6	5	5	5	6	6	6	4	4	5	5	6	7	4	3	1	2	5	6	6	4	4	2	3	2	2	2	2	2	2	4	3	3	1
42	31	5941582	422	12	1	1	2	5	5	4	1	5	1	1				7	5	6	7	7	6	5	6	6	6	6	5	5	6	7	6	1	6	1	4	4	1	3	2	5	1	1	5	5	3	3	1	1	3	3	5	6	1	5
43	31	5941582	5	12	1	1	1	6	5	7	1	3	3	1	4	4	8	7	7	7	7	7	7	7	7	7	7	7	7	7	7	7	7	7	7	1	1	1	1	1	1	7	1	1	3	3	1	1	1	1	1	1	7	7	1	4
44	31	5941582	614	12	1	1	2	6	5	3	1	6	3	1				5	2	5	7	2	4	4	5	5	6	5	5	4	4	5	3	6	6	2	1	1	1	1	1	7	5	5	2	3	2	1	1	1	2	1	2	1	4	4
45	31	5941582	659	12	1	1	2	6	1	6	1	3	3	1				7	7	7	5	7	6	6	6	6	4	4	4	4	4	4	4	2	2	3	5	4	2	2	2	2	2	2	4	4	2	2	1	1	1	1	1	1	1	3
46	31	5941582	68	12	1	1	2	5	1	4	1	7	3	1				7	7	7	4	5	2	6	6	7	4	4	4	7	7	7	6	1	7	1	2	2	1	1	7	7	1	1	7	7	6	4	1	2	2	2	7	7	4	6
47	31	5941582	1164	12	1	1	2	6	5	4	1	6	1	1				7	7	1	7	7	7	1	1	1	1	4	6	1	1	7	4	4	4	1	1	1	1	7	7	4	4	4	1	1	1	1	1	1	1	1	1	1	1	1
48	31	5941582	495	12	1	1	2	4	5	6	1	4	3	1				7	7	7	7	7	7	7	7	7	7	7	7	7	7	7	7	1	7	1	1	1	1	1	1	7	1	1	5	5	1	1	1	1	2	2	7	7	1	4
49	31	5941582	542	12	1	1	2	5	1	5	1	6	1	1				1	1	1	4	1	1	1	1	1	4	4	3	1	1	5	5	1	7	1	1	1	1	7	7	7	1	5	1	4	1	1	1	1	1	4	1	1	1	1
5	31	5941582	88	12	1	1	1	5	5	4	1	6	3	2	1	29		4	3	3	6	6	6	5	4	5	7	6	7	7	7	7	7	7	7	2	2	1	1	2	1	7	2	2	6	5	3	3	2	2	3	3	5	3	3	3
51	31	5941582	345	12	1	1	2	3	2	7	1	5	3	1				1	1	1	1	1	1	1	4	4	4	4	4	1	1	1	1	1	4	1	3	7	4	4	4	4	4	4	4	4	4	4	5	4	4	4	4	4	5	4
52	31	5941582	36	12	3	1	2	5	5	4	1	5	3	1				6	1	1	1	1	1	1	1	1	1	1	1	1	1	1	1	1	1	1	1	5	6	7	7	1	7	7	4	4	7	5	2	7	4	7	7	7	1	1
53	31	5941582	618	12	1	1	1	5	5	4	3	6	3	1	1	33		4	3	3	6	5	5	6	6	5	3	4	5	4	4	4	5	7	7	3	2	1	5	4	4	5	4	4	1	2	3	4	1	2	1	3	4	3	4	5
54	31	5941582	333	12	1	1	2	4	5	3	1	6	3	1				7	6	7	7	7	7	7	7	7	7	7	7	7	7	7	7	6	6	1	1	1	1	1	1	7	2	2	1	1	1	1	1	1	1	1	7	4	1	7
55	31	5941582	818	12	1	1	2	6	5	3	1	4	5	1				7	7	7	7	7	7	6	7	7	7	7	7	7	7	7	7	7	7	1	1	1	1	1	1	7	1	1	6	6	1	1	1	1	1	1	6	7	1	2
56	31	5941582	535	12	1	1	2	7	5	7	1	5	3	1				7	7	7	7	7	7	7	7	6	7	6	6	7	7	7	7	5	5	2	7	1	1	1	1	7	2	1	3	3	1	1	1	1	1	3	7	7	3	3

57	31	5941582	318	12	1	1	2	5	5	6	1	6	5	1				7	7	7	7	5	5	4	4	4	7	4	7	5	5	7	7	4	4	1	1	1	1	1	6	1	1	1	1	1	1	1	1	1	1	1	1	3	1	5
58	31	5941582	1754	12	1	1	2	7	5	4	1	3	3	1				5	5	4	4	5	5	4	3	4	5	4	4	5	5	2	2	4	2	5	4	5	3	4	2	5	5	4	7	7	2	2	3	5	6	6	6	7	6	2
59	31	5941582	498	12	1	1	2	5	1	6	1	7	1	1				5	4	4	7	4	4	4	3	4	4	4	4	5	5	5	5	2	5	2	5	4	2	4	3	6	4	4	6	6	5	3	4	6	4	3	5	6	2	5
6	31	5941582	546	12	1	1	2	5	5	7	1	7	1	1				3	1	1	5	6	2	3	3	3	5	7	3	2	2	3	2	1	3	2	2	2	6	7	4	6	4	4	1	2	2	2	1	2	4	2	5	3	6	3
61	31	5941582	285	12	1	1	2	3	1	6	1	5	3	1				6	5	6	7	7	7	4	4	4	7	7	7	6	6	6	6	7	7	2	2	1	2	3	3	6	3	2	1	1	2	4	1	1	1	1	4	4	3	5
62	31	5941582	592	12	1	1	2	7	5	3	1	3	3	1				7	7	7	7	7	7	7	7	7	7	7	7	7	7	7	7	7	7	1	1	1	1	1	1	7	1	1	3	3	1	1	1	1	1	3	5	5	2	3
63	31	5941582	475	12	1	2	1	7	1	7	1	2	1	2	4	35	8	7	6	7	7	7	7	6	5	5	4	4	4	7	7	7	7	2	7	1	1	1	1	2	1	7	2	2	2	2	1	1	1	2	2	2	7	6	2	7
64	31	5941582	776	12	1	1	1	3	5	6	1	6	3	1	1	4		3	3	3	7	7	5	7	7	7	7	7	4	7	7	7	7	6	6	2	1	1	1	1	1	7	1	5	1	1	1	1	1	1	1	1	6	5	2	6
65	31	5941582	384	12	1	1	2	2	5	3	1	6	5	1				6	7	7	7	6	6	6	7	6	6	6	5	7	7	7	7	7	7	1	1	1	1	4	2	6	5	5	1	2	2	1	1	1	1	1	3	2	1	6
66	31	5941582	565	12	1	1	2	4	5	6	1	6	5	1				7	6	6	7	5	5	7	7	7	6	6	6	6	6	7	7	6	6	1	1	1	1	2	2	7	2	2	2	2	2	2	1	1	1	1	4	4	2	3
67	32	5941582	-1	12	1	1	2	4	1	6	1	6	3	1				7	6	6	7	5	5	6	6	6	4	6	5	6	6	7	7	5	7	2	2	2	2	2	1	6	2	2	2	3	2	2	1	2	1	2	5	5	3	5
68	31	5941582	67	12	4	1	2	4	1	6	1	6	3	1				2	1	2	2	1	1	3	3	3	2	2	2	1	1	4	1	7	4	6	1	1	1	7	7	7	1	1	4	4	1	1	1	1	1	1	1	1	1	2
69	31	5941582	433	12	1	1	2	3	5	6	1	6	3	1				7	6	6	7	6	6	3	3	3	6	6	6	7	6	6	5	4	4	6	5	5	2	3	4	3	4	4	1	1	5	4	2	1	1	4	5	5	2	2
7	31	5941582	59	12	1	1	1	6	1	6	1	6	3	1	3	15	9	6	6	7	7	7	6	6	6	6	7	7	7	7	7	6	6	5	6	1	1	1	2	2	7	7	2	2	3	3	1	1	1	1	1	1	4	3	1	6
71	31	5941582	162	12	3	1	2	5	5	8	1	6	1	1				1	4	1	4	4	4	4	4	4	2	3	1	1	1	4	4	1	1	7	7	7	1	7	3	1	3	3	5	4	3	3	3	4	4	3	7	7	3	3
72	31	5941582	392	12	1	1	1	6	5	4	2	6	1	1	1	18	18	6	6	6	6	6	7	2	1	2	3	3	3	5	5	6	6	7	4	1	5	1	2	2	5	5	3	2	3	4	2	2	4	6	4	4	4	4	2	4
73	31	5941582	8	12	1	1	2	6	1	3	1	3	3	1				3	3	2	3	5	2	3	3	3	4	3	3	1	1	4	3	7	7	3	3	1	2	2	2	6	3	3	1	1	1	1	1	1		1	2	2		2
74	31	5941582	654	12	1	1	1	5	5	4	2	6	3	1	1	6	17	7	7	7	7	7	7	5	5	4	7	6	7	7	7	7	7	5	7	2	1	1	1	2	2	7	1	1	1	1	1	1	1	1	1	1	4	2	1	7
75	31	5941582	477	12	1	2	2	7	1	6	1	3	3	1				7	7	6	7	7	7	5	5	5	7	6	7	7	7	6	6	7	7	5	7	1	1	1	1	7	1	1	3	1	1	1	1	1	1	1	6	7	1	7
76	31	5941582	613	12	1	1	2	6	1	6	1	3	1	2				7	4	4	7	7	7	7		7				7	7	7	7	7	7	1	1	1	1	1	2	6	2	3	1	5	1	1	1	1	1	1	3	1	1	6
77	31	5941582	577	12	1	1	2	4	1	6	1	6	3	1				6	6	7	7	4	4	6	6	6	6	7	5	6	5	6	6	3	4	1	3	1	1	1	2	6	3	3	1	1	5	4	1	2	3	1	5	3	1	2
78	31	5941582	551	12	1	1	2	6	5	4	1	4	3	1				7	7	7	7	7	7	7	7	7	7	7	7	7	7	7	7	7	7	1	7	1	1	1	1	7	1	1	4	5	2	2	1	1	1	1	7	7	2	2
79	31	5941582	791	12	1	1	2	6	5	4	1	3	3	2				7	7	7	7	7	6	6	7	7	6	6	6	7	7	7	6	7	7	6	2	1	1	1	1	7	1	1	6	5	6	4	4	4	4	4	4	5	4	4
8	31	5941582	358	12	1	1	1	6	1	4	1	3	3	2	3	45		7	6	5	6	6	6	7	6	6	6	6	6	6	6	5	6	6	6	1	1	1	1	1	1	7	2	2	2	2	1	1	1	1	2	2	4	3	3	2
81	31	5941582	1147	12	4	1	2	7	1	8	3	5	3	1				1	2	1	1	4	3	1	1	1	1	1	1	1	1	1	3	1	1	7	7	7	7	7	7	1	6	6	1	1	2	1	1	1	2	1	4	2	3	1
82	31	5941582	567	11	3	1	2	3	1	7	6	5	1	2				3	4	4	1		2	6	5	6	4	5	4	1	1	4	4	7	7	6	1	1	1	7	1	5	3	3	1	1	4	1	1	1	1	1	4	5	1	5
83	31	5941582	1288	12	1	1	2	6	1	6	1	5	1	1				6	6	6	5	6	5	1	1	2	5	5	5	6	6	7	5	7	7	1	1	1	1	3	2	7	1	2	1	1	1	1	1	1	1	1	4	2	1	2
84	31	5941582	452	12	1	1	2	6	1	6	1	3	3	1				2	2	2	2	6	6	6	6	6	3	3	3	5	5	5	6	6	6	2	2	2	2	6	2	6	1	1	1	1	1	1	1	1	1	1	6	6	1	1
85	31	5941582	8	12	1	1	1	5	5	6	1	6	1	1	3	36		7	7	7	6	7	6	6	6	6				7	7	6	4	5	6	1	1	1	1	1	2	7	3	4	3	3	1	1	1	1	2	1	4	4	2	
86	31	5941582	1181	12	1	1	2	7	5	3	1	3	3	2				7	7	7	7	5	5	7	7	7	7	7	7	7	7	7	7	3	7	1	1	1	1	1	1	7	5	2	3	5	1	1	1	1	1	1	1	1	1	1

87	31	5941582	514	12	1	1	2	2	5	3	1	6	3	1				5	5	3	6	6	2	4	3	4	2	2	2	5	5	5	2	6	6	3	1	1	2	2	3	6	7	7	2	2	2	2	2	6	2	3	5	4	3	5
88	31	5941582	584	12	1	1	2	3	5	6	1	6	5	1				6	5	5	6	6	6	6	6	6	7	7	4	6	6	5	5	5	5	1	1	1	1	7	5	7	2	2	1	1	1	1	1	1	1	1	3	2	3	5
89	32	5941582	-1	12	1	1	2	6	5	6	1	3	3	1				5	2	4	5	7	7	7	7	7	1	1	1	4	4	3	2	5	7	1	1	1	1	5	1	7	2	2	2	2	1	2	1	1	2	1	3	3	4	2
9	31	5941582	5	12	1	1	2	6	5	6	1	4	3	1				5	4	4	6	5	4	3	4	4	6	4	6	5	5	4	4	7	5	5	2	2	3	4	4	6	2	2	2	2	2	2	2	2	4	2	3	1	1	4
91	31	5941582	1742	12	5	1	1	6	1	4	5	6	4	2	3	44	8	7	6	7	7	6	6	7	6	6	6	6	5	7	6	7	7	7	7	1	2	1	2	2	2	7	3	4	5	6	5	6	4	6	5	2	6	7	4	3
92	31	5941582	457	12	1	1	2	6	1	6	1	3	3	1				7	7	7	6	7	7	6	7	7	4	4	3	7	7	7	6	7	7	1	1	1	1	1	1	7	2	2	1	1	1	1	1	1	1	1	6	6	2	6
93	31	5941582	145	12	1	1	2	6	1	9	3	5	2	1				7	7	7	7	7	7							7		7	7	4	4			4						4	4	4	1	4	1	1	4	4	7	7	1	1
94	32	5941582	-1	12	1	1	2	6	5	4	1	3	1	1				7	6	7	7	7	7	6	6	6	6	6	6	7	7	7	7	6	6	2	6	2	1	1	2	6	2	2	2	2	2	2	2	2	2	2	6	4	2	2
95	31	5941582	614	12	1	1	2	7	5	6	1	2	3	1				7	7	7	7	7	7	7	7	7	7	7	7	7	7	7	7	7	7	1	1	1	1	1	1	7	2	2	3	5	1	1	1	1	2	1	3	4	2	3
96	31	5941582	524	12	1	1	1	4	5	8	1	6	3	1	1	1		7	6	7	6	4	3	5	5	5	7	7	6	7	7	5	4	4	6	2	1	1	1	2	1	7	4	4	3	3	1	1	1	2	2	2	6	2	2	1
97	31	5941582	147	12	1	1	2	6	1	8	1	2	3	1				7	1	1	1	1	1	7	7	6	1	4	2	1	1	1	1	7	6	2	1	1	2	7	2	2		4	3	2	1	4	1	4	6	7	7	7	4	6
98	31	5941582	1573	12	1	1	2	6	5	9	1	3	3	1				3	3	3	2	4	3	1	1	1	3	3	3	3	3	3	3	3	3																					
99	31	5941582	165	12	1	1	2	5	1	4	1	5	3	2				6	6	6	4	5	5	4	4	5	4	4	4	6	6	7	6	3	4	4	5	3	1	1	1	7	4	4	5	5	5	6	2	2	6	2	6	5	2	5
1	31	5941582	3294	12	1	1	2	5	5	4	1	5	3	1				7	7	7	7	7	7	7	7	7	4	7	4	7	7	7	7	1	7	1	1	1	1	1	1	7	4	4	6	6	6	6	4	4	7	5	7	7	1	6
11	31	5941582	844	12	1	1	2	3	5	6	1	6	5	1				6	4	5	6	6	6	6	5	6	7	6	7	6	6	6	6	5	4	1	1	1	2	3	2	7	1	1	1	1	1	1	1	1	1	1	5	5	1	4
12	31	5941582	566	12	1	1	1	7	5	4	1	4	1	1	3	4		7	6	6	7	7	7	5	6	6	7	7	7	7	7	7		7	7	1	1	1	1	1	1	7	7	7	1	1	1	1	1	1	1	1	7	7	1	1
13	31	5941582	581	12	1	1	2		1	6	1	3	5	1				2	2	2	6	4	4	1	2	1	2	2	2	2	6	6	6	7	7	3	2	1	5	7	7	5	2	2	2	1	1	1	1	1	2	2	2	3	2	1
14	31	5941582	524	12	3	1	2	3	5	6	1	6	7	1				1	5	5	3	4	4	3	3	3	3	3	3	3	3	1	2	1	3	5	3	5	2	6	5	2	1	1	1	1	1	1	1	1	1	1	1	1	1	1
15	31	5941582	1483	12	1	1	2	5	5	6	1	4	3	1				7	7	7	7	7	7	7	7	7	7	7	7	7	7	7	7	7	7	1	1	1	1	1	1	7	1	1	1	1	1	1	1	2	1	1	2	2	1	1
16	31	5941582	123	12	1	1	2	7	1	7	5	2	3	1				7	7	7	7	7	7	7	7		7	7	7	7	7	7	7	5	4	1	7	1									1	1	1	1	2	2	7	7	1	7
17	31	5941582	311	12	1	1	2	3	5	6	1	7	3	1				7	5	6	6	4	5	6	6	6	5	5	4	7	7	7	6	5	5	3	3	2	2	1	1	6	4	4	2	2	7	5	2	5	4	6	7	7	4	2
18	31	5941582	1329	12	1	1	2	7	1	6	1	2	3	1				6	1	1	7	7	6	5	5	5				1	1	3	3	1	1	1	4	5	1	7	2	4	1	1	3	3	1	1	1	1	1	6	5	5	1	1
19	31	5941582	169	12	1	6	2	6	5	3	5	4	3	1				7	1	1	7	1	7	1	1	1	4	4	4	1	1	2	2	1	1	7	2	4	7	7	7	4	1	1	4	7	1	1	3	4	4	4	7	7	2	4
11	31	5941582	1342	12	1	1	2	7	1	4	4	2	3	1				7	7	7	7	5	7	4	5	5	4	4	3	7	5	6	5	5	5	3	4	4	5	7	5	4	4	4	5	6	2	2	5	2	3	3	7	7	4	3
111	31	5941582	518	12	1	1	2	4	5	7	1	7	1	1				7	6	7	7	7	6	7	7	7	7	7	7	7	7	5	4	4	6	1	1	1	1	1	1	7	2	2	2	2	2	3	1	1	2	1	5	5	2	2
112	31	5941582	455	12	1	1	2	2	5	5	1	5	3	1				2	2	3	1	2	3	6	5	6	5	5	5	5	6	5	4	7	7	2	2	1	2	4	3	7	1	1	2	1	1	1	1	1	1	2	3	4	2	5
113	31	5941582	48	12	1	1	2	3	5	6	1	4	5	1				7	4	6	4	5	5	3	3	3	5	5	5	4	4	4	3	7	7	1	2	1	2	2	5	6	3	3	2	2	2	2	1	1	2	2	3	3	3	3
114	31	5941582	842	12	1	1	2	5	5	6	1	6	1	3				5	4	7	7	6	6	7	7	7				7	7	7	7	3	4	2	3	1	1	1	1	7			3	4	4	4	2	3	2	2	6	5	1	5
115	31	5941582	39	12	1	1	2	5	5	6	1	6	6	1				4	2	2	5	4	4	3	1	3	5	5	4	2	3	7	5	2	3	4	6	3	2	3	3	5	4	4	5	5	2	2	2	2	2	2	4	3	2	2
116	31	5941582	141	12	1	1	2	6	5	4	1	5	3	1				7	4	4	7	7	7	3	3	3	5	5	5	6	6	7	7	5	6	1	1	1	3	3	2	6	2	4	5	3	1	2	2	5	3	2	5	6	2	4

117	31	5941582	712	12	1	1	2	6	5	6	1	4	3	1				7				7	7	5	5	5	6	6	6	7	6	5	4	7	7	2	2	1	1	1	2	6	4	4	1	1	1	1	1	1	6	2	4	4	2	2
118	31	5941582	466	12	1	1	2	5	5	6	1	6	3	1				5	3	6	7	4	7	6	6	6	6	6	6	6	6	6	4	4	4	1	2	1	1	1	1	7	4	4	3	3	4	2	2	2	2	2	6	4	3	3
119	31	5941582	449	12	1	1	2	5	5	4	1	4	3	1				5	3	5	7	5	5	1	1	1	3	3	1	5	3	7	7	7	7	1	1	1	1	7	1	7	1	1	1	1	7	1	1	1	1	1	1	7	1	3
12	31	5941582	493	12	1	1	2	2	5	3	3	7	2	1				3	6	5	4	2	1	7	7	7	4	5	6	6	7	7	5	5	7	1	1	1	2	2	2	6	4	3	1	1	2	2	1	1	1	1	5	2	3	6
121	31	5941582	747	12	1	1	2	6	5	3	1	3	3	1				7	7	7	7	7	7	5	5	5	6	6	7	7	7	7	7	7	7	1	1	1	1	1	1	7	1	1	1	1	1	1	1	1	1	1	3	3	1	1
122	31	5941582	341	12	1	1	2	4	5	6	1	6	3	1				5	5	5	7	6	5	6	6	4	7	7	7	7	7	7	5	6	6	1	1	1	1	1	1	7	6	6	3	4	3	1	1	1	3	2	5	5	3	1
123	31	5941582	229	12	1	1	2	6	5	8	1	5	1	1				7	7	7	2	7	2	4	7	6	5	5	4	4	4	5	7	1	1	7	7	7	4	1	7	5	1	1	7	7	7	7	4	7	7	7	7	7	3	4
124	31	5941582	969	12	1	1	2	5	5	3	1	7	1	1				1	3	4	7	7	6	7	7	7	7	4	2	3	3	6	3	3	3	4	4	3	7	7	4	5	5	5	2	4	4	3	1	2	2	4	5	4	3	5
125	31	5941582	416	12	1	1	2	6	5	3	1	6	1	1				7	5	3	7	3	3	2	3	2	4	3	3	3	5	5	3	5	5	1	2	1	3	5	5	6	2	2	5	6	2	2	2	1	3	3	7	3	2	3
126	31	5941582	1898	12	1	1	2	6	5	3	1	3	3	2				7	4	4	7	4	4	6	6	6	7	7	7	7	7	7	7	7	7	1	1	1	1	1	1	7	7	1	2	4	1	1	1	1	3	3	4	4	1	4
127	31	5941582	656	12	1	1	2	6	5	4	1	6	6	1				7	2	6	7	6	6	7		6	7			6	6	5	5	6	6	1	1	1	2	3	2		4	4	5	7	1	1	1	4	5	5	5	6	1	1
128	31	5941582	526	12	4	1	2	2	5	3	1	6	6	2				4	1	4	1	1	1	1	1	1	1	1	1	1	1	1	1	7	6	1	1	1	4	7	7	4	2	1	1	1	1	1	1	1	2	1	1	1	1	1
129	31	5941582	651	12	1	1	1	3	5	5	1	6	3	1	1	4		7	3	6	3	7	5	7	3	7	3	3	3	7	7	7	4	7	5	1	2	1	1	1	1	6	1	1	1	1	1	1	1	1	1	2	6	6	1	7
13	31	5941582	1413	12	1	1	2	6	5	4	1	3	3	1				7	7	7	7	7	7	7	7	7	7	7	7	7	7	7	5	4	7	1	1	1	1	1	1	7	5	5	7	7	2	2	1	1	2	1	7	4	1	3
131	31	5941582	412	11	1	1	2	5	5	6	1	5	1	1				4	1	3	2	5	4	7	7	7	1	1	1	2	2	1	5	6	4	1	2	1	1	4	1	7	1	1	1	2	1	1	1	1	1	1	2	4	1	1
132	31	5941582	466	12	1	1	2	6	5	3	1	3	3	1				7	6	6	7	6	6	5	5	5	6	6	7	7	7	7	7	7	4	2	1	4	1	1	1	7	4	6	6	6	7	7	2	6	7	5	7	7	5	6
133	31	5941582	157	12	1	1	2	6	5	6	1	4	3	1				3	3	3	2	2	2	3	3	3	4	4	4	3	4	2	3	5	2	6	5	4	5	7	4	5	3	4	2	2	1	1	1	1	1	2	6	3	1	1
134	31	5941582	595	12	1	1	1	6	5	6	1	6	3	1	3	35	1	7	7	7	7	7	7	7	7	7	7	7	7	7	7	7	7		7	1	1	1	1	1	1	7	1	1	4	4	1	1	1	1	1	1	5	5	1	7
135	31	5941582	869	12	1	1	2	6	5	3	1	3	1	1				5	5	5	7	7	6	5	2	4	4	2	4	5	6	7	7	5	5	1	1	1	1	1	6	7	6	7	5	7	1	1	1	1	1	1	3	2	1	3
136	31	5941582	46	12	1	1	2		5	3	1	5	3	1				7	6	7	7	7	3	4	4	5	7	5	5	7	7	7	6	7	7	6	1	1	1	1	2	7	3	3	1	7	1	1	1	1	1	1	7	7	1	4
137	31	5941582	645	12	3	1	2	6	5	5	6	4	3	2				2	1	1	1	1	1	4	4	5	2	2	3	1	1	2	3	1	1	7	6	2	1	7	2	6	4	4	2	2	2	2	2	4	2	2	4	4	3	2
138	32	5941582	-1	12	3	1	2	4	5	6	1	5	3	1				7	5	5	5	6	6	6	5	6	7	7	7	7	7	7	6	6	6	1	1	1	1	1	1	7	2	2	1	1	1	1	1	1	1	1	2	1	1	1
139	31	5941582	216	12	1	1	2	2	1	3	1	4	3	1				6	6	5	6	4	4	6	5	5	4	5	4	5	5	6	4	4	4	2	1	3	2	2	2	6	2	2	2	3	1	2	1	2	2	1	2	2	2	4
14	31	5941582	327	12	1	1	2	7	1	4	1	3	3	2				6	5	6	6	6	6	5	3	5	6	5	5	6	6	5	5	5	5	1	1	1	2		2	7	4	4	5	5	1	1	3	3	3	2	4	3	4	4
141	31	5941582	943	12	1	1	2	6	1	4	1	4	1	1				7	6	7	7	7	7	7		7	6	6		7	7	7	7	7	7	1	7	1	1	1	1	7	5	5	5	6	7	3	4	5	5	5	7	7	4	3
142	31	5941582	765	12	1	1	2	3	5	6	1	5	3	1				6	5	5	7	7	7	7	7	7	1	1	1	6	6	7		6	7	1	1	1	1	1	2	7	4	5	3	2	1	1	1	1	2	1	6	3	3	5
143	31	5941582	716	12	1	1	2	5	5	7	1	6	3	1				7	7	7	7	7	5	6	6	7	7	7	7	7	7	7	7	7	7	1	1	1	2	2	1	7	4	4	3	3	2	1	1	1	1	1	1	1	1	1
144	31	5941582	726	12	1	4	2	4	1	4	6	1	7	1				7	5	5	4	5	5	5	5	5	5	3	3	5	5	5	4	5	5	1	1	1	1	3	2	6	2	2	2	2	2	2	1	1	2	2	6	5	2	2
145	31	5941582	57	12	1	1	2	1	5	2	6	8	7	3				7	4	1	7	7	6	5	7	7	7	7	7	7	7	7	4	7	7	1	1	1	1	1	1	7	1	1	1	1	1	1	1	1	1	1	5	2	3	1
146	31	5941582	438	12	1	1	2	5	1	6	1	4	1	1				6	4	7	7	7	4	1	1	1	6	4	4	5	5	6	5	6	6	1	1	1	1	1	3	7	4	2	1	4	1	1	1	1	1	1	5	4	1	4

147	31	5941582	141	12	1	1	2	2	5	3	1	7	1	2				7	6	7	6	6	6	7	7	7	4	4	4	6	7	7	7	4	7	1	1	1	1	1	1	7	1	1	1	2	1	1	1	1	1	1	1	4	1	7
148	31	5941582	928	12	1	1	2	5	5	6	1	4	3	1				7	1	7	7	4	6	7	7	7	7			7	7	7	7	5	5	1	5	1	1	1	1	7	2	2	4	4	4	3	3	4	4	3	5	3	5	6
149	31	5941582	64	12	1	1	2	4	5	4	1	6	3	1				6	3	3	3	5	5	5	5	5	5	6	6	6	5	6	6	4	4	2	2	2	3	3	2	6	5	5	5	5	4	2	3	5	5	3	6	6	2	4
15	31	5941582	37	12	1	1	2	4	5	7	1	5	3	1				5	5	5	6	4	3	5	5	5	1	1	1	5	6	5	3	6	6	2	4	2	3	5	3	6	5	5	3	3	2	2	1	1	4	4	2	2	2	1
151	31	5941582	67	12	1	1	2	3	1	4	1	7	3	1				7	7	7	7	7	7	7	7	7	3	3	3	7	7	7	7	4	4	1	1	1	1	1	1	7	4	4	4	4	7	4	1	1	1	1	4	5	1	4
152	31	5941582	584	12	1	1	2	3	5	6	1	6	5	1				6	6	6	6	6	7	6	6	6	6	6	6	5	5	7	7	6	6	1	1	1	1	2	1	7	2	2	4	4	1	1	2	2	2	3	4	4	2	2
153	31	5941582	396	12	1	1	1	3	1	4	1	6	1	1	2	1	6	7	5	6	7	6	6	6	4	5	5	5	6	6	4	4	4	5	5	3	6	5	5	3	3	3	3													
154	31	5941582	34	12	1	1	2	1	1	3	1	8	1	1				7	6	2	7	2	2	6	6	7	5	1	1	6	7	7	5	2	7	1	1	1	2	1	2	2	1	1	1	1	1	1	1	1	1	1	4	5	2	1
155	31	5941582	518	12	1	1	2	4	1	5	1	5	3	1				6	7	6	7	7	7	7	7	7	4	4	4	7	7	7	7	7	7	1	1	1	1	1	2	7	1	1	2	2	1	1	1	1	1	2	4	3	2	1
156	31	5941582	596	12	1	1	2	6	1	4	1	5	3	1				2	2	4	7	4	2	4	3	4	1	2	4	6	4	1	1	4	4	1	4	4	4	3	4	4	1	1	3	3	1	5	1	1	1	4	2	2	1	4
157	31	5941582	785	12	1	1	1	5	1	6	1	6	5	1	1	7		2	1	7	1	1	1	1	1	1	1	5	5	1	1	1	1	2	2	7	7	7	7	7	3	2	5	5	7	7	1	7	7	7	7	7	7	2	7	7
158	31	5941582	1333	12	1	1	2	2	5	3	1	7	1	1				6	7	7	7	7	7	6	5	6	6	7	4	7	7	7	6	7	7		2	4	1	2	1	7	4	4	5	4	5	7	7	7	7	3	6	2	3	6
159	32	5941582	-1	12	1	1	2	5	5	6	1	5	1	1				6	6	5	5	7	6	5	5	5	7	7	7	6	6	7	7	7	7	1	1	1	1	2	1	7	2	2	1	1	1	1	1	1	1	1	6	3	1	7
16	31	5941582	41	12	1	1	2	2	1	3	1	5	3	1				7	5	3	7	6	7	7	5	6	4	6	5	6	6	7	7	4	4	2	2	1	2	2	2	7	6	3	1	2	5	1	1	1	2	5	7	6	2	6
161	31	5941582	125	12	1	1	1	2	5	3	1	1	3	2	1	2		7	7	7	7	6	5	4	3	3	6	6	6	7	7	7	5	7	6	1	1	1	1	1	1	7	3	4	1	2	1	1	1	1	1	1	1	1	1	1
162	31	5941582	54	12	1	1	2	6	5	3	2	2	3	1				4	4	4	4	6	4	4	4	4	4	4	4	6	5	6	4	3	2	4	4	7	4	4	4	4	4	4	5	6	4	3	2	3	4	3	4	5	4	4
163	31	5941582	6	12	1	1	2	2	5	3	1	7	3	1				7	3	5	6	3	6	3	2	2	6	5	4	7	7	7	4	7	7	1	2	2	1	5	2	7	3	3	1	1	1	1	1	1	1	2	1	3	1	1
164	31	5941582	279	12	1	1	2	2	5	3	1	6	1	1				7	7	7	7	6	7	7	6	5	4	4	6	7	7	7	7	1	7	1	1	1	1	1	1	7	4	4	2	2	4	4	1	1	2	1	6	5	4	4
165	31	5941582	543	12	1	1	2	2	1	3	1	6	3	1				7	5	4	3	2	2	6	6	6	5	5	6	5	5	5	5	7	7	1	1	1	1	3	1	7	5	5	1	1	1	3	1	1	1	1	4	3	1	1
166	31	5941582	588	12	1	1	1	3	5	4	1	6	3	1	1	1	5	6	7	7	7	7	5	7	7	7	7	7	7	7	7	7	5	7	7	1	1	1	2	1	1	7	6	2	1	1	1	1	2	2	2	2	7	1	1	7
167	31	5941582	636	12	1	6	2	3	5	3	2	7	1	2				7	7	7	7	7	7	7	7	7	7	7	5	7	7	7	7	7	7	1	1	1	1	1	5	7	3	2	1	1	1	1	1	1	1	1	7	7	4	5
168	31	5941582	178	12	1	1	2	6	5	6	1	4	3	1				4	2	2	6	3	5	6	6	6	6	4	5	6	5	6	6	6	6	2	2	2			6	6	5	5	4	3	3	3	2	2	2	2	5	6	3	3
169	32	5941582	-1	12	1	1	2	7	5	4	1	3	1	1				7	7	7	7	7	7	7	7	7	7	7	7	7	7	7	7	7	7	7	7	7	1	1	1	1	4	4	6	6	1	1	1	4	1	1	7	4	1	7
17	31	5941582	859	12	1	1	2	4	5	5	1	5	1	1				7	7	7	7	7	7	7	6	6	7	7	7	7	7	7	7	4	5	1	1	2	1	1	1	7	3	3	4	5	3	3	5	7	6	6	7	7	5	7
171	31	5941582	1354	12	1	1	2	5	1	6	1	6	5	1				2	2	1	6	5	6	3	3	3	1	1	1	1	1	2	1	6	6	6	4	4	2	6	7	5	4	4	2	2	2	2	2	2	3	2	4	4	2	2
172	31	5941582	392	12	1	1	2	3	1	6	1	6	3	1				2	6	7	5	4	5	7	7	7	7	7	1	4	3	2	2	7	7	1	1	1	1	1	1	7	5	6	3	2	2	1	1	1	1	1	5	5	1	1
173	31	5941582	58	12	1	1	2	5	1	3	1	7	1	1				6	7	6	7	7	7	6	6	6	6	6	6	6	6	7	7	6	6	1	1	1	2	2	2	7	2	2	2	2	2	2	1	1	2	2	7	7	2	2
174	32	5941582	-1	12	1	1	2	8	1	2	2	1	3	1				7	6	5	6	7	7	5	6	6	4	7	4	7	7	7	7	6	6	2	1	1	2	1	1	7	4	4	5	5	2	4	3	2	4	2	5	5	3	5
175	31	5941582	342	12	1	1	2	5	1	5	1	7	6	1				2	2	2	2	2	3	3	3	3	2	3	2	3	3	3	2	5	6	4	3	4	6	7	5	4	5	5	2	2	3	3	2	4	2	3	4	4	3	3
176	31	5941582	546	12	1	1	2	5	1	3	1	5	1	1				7	7	7	7	7	7	5	5	5	6	7	7	7	7	7	7	7	7	1	1	1	1	2	1	7	1	1	1	1	1	1	1	1	1	1	1	1	1	1

177	31	5941582	253			1	2		1	6	1	6	3					5	3	3	6	6	6	7	7	7	4	4	4	6	7	7	5	7	7	1	7	1	1	1	1	7	4	4	1	1	1	1	1	1	1	1	4	2		1
178	31	5941582	1483			1	1		5	2	2	5	2		1	5	4	1	1	1	1	1	1				1	1	1	1	1	1	1	7	1	1	1	1	7	7	7	7	1	1	7	7	1	1	1	1	1	2	4	6	3	3
179	31	5941582	1515			1	1		5	1	1	1	1		1	8	8	1	1	1	1	1	1	1	1	1	1	1	1	1	1	1	1	1	1	1	1	1	7	7	7	7	1	1	7	7	1	1	1	1	1	1	1	1	1	1
18	31	5941582	695			1	2		2	2	1	1	3					7	7	7	7	7	7	7	7	7	7	7	7		1	1	1	1	1	1	1	1	7	7	7	7	1	1	7	7	1	1	1	1			1	1	1	1
181	31	5941582	1458			3	1		1	6	1	6	4		1	16	5	4	4	4	4	4	4	4	4	4	4	4	4	4	4	4	4	4	4	4	4	4	4	4	4	4	4	4	4	4	4	4	4	4	4	4	4	4	4	4
182	31	5941582	54			1	1		1	6	1	6	1		1	3	6	4	4	4	4	4	4	4	4	4	4	4	4	4	4	4	4	4	4	4	4	4	4	4	4	4	4	4	4	4	4	4	4	4	4	4	4	4	4	4
183	31	5941582	214			1	1		5	3	2	4	1		1	78	12	6	5	3	5	4	4	4	4	4	6	4	5	5	5	5	5	5	5	5	5	5	3	3	3	3	5	5	2	2	4	4	4	4	4	4	5	5	5	5
184	31	5941582	246	12	1	1	2	5	5	6	1	4	3	1				7	7	7	7	7	7	5	4	4	4	5	5	7	7	7	7	7	7	1	1	1	1	1	1	7	1	1	1	1	1	1	1	1	1	1	5	2	1	1
185	31	5941582	358	12	1	1	2	2	1	5	1	6	6	1				7	5	4	7	7	4	5	5	5	5	7	3	3	3	7	5	4	7	2	1	1	1	3	5	7	1	1	1	1	1	1	1	1	2	1	4	3	2	4
186	31	5941582	458	12	1	1	2	5	5	6	1	6	1	1				7	6	6	7	7	7	7	7	7	7	4	4	7	7	7	7	7	7	1	1	1	1	1	1	7	1	1	1	1	1	1	1	1	1	1	1	1	1	1
187	31	5941582	623	12	3	1	2	5	5	3	1	5	1	1				5	5	4	4	4	4	4	4	4	4	3	4	4	4	4	4	3	3	2	2	2	3	3	4	5	4	3	4	5	4	3	2	2	4	4	6	5	2	3
188	31	5941582	351	12	1	1	2	3	5	3	1	4	3	1				7	5	6	7	4	4		5	5	4	4	4	7	7	4	5	4	7	1	1	1	1	1	1	7	4	4	1	1	1	1	1	1	1	1	4	4	1	1
189	31	5941582	752	12	1	1	2	5	5	6	1	5	3	1				7	7	7	7	7	7	5	5	5	6	5	6	7	5	5	5	5	3	1	1	1	1	1	1	7	4	4	5	6	2	3	2	2	3	3	6	5	3	5
19	31	5941582	37	12	1	1	2	3	5	6	1	6	6	1				6	5	5	4	6	5	2	2	2	5	6	6	6	6	6	6	4	4	2	5	2	1	4	2	6	3	3	3	3	2	2	2	2	2	2	3	3	2	5
191	31	5941582	47	12	1	1	1	4	5	4	1	6	1	1	3	26	7	7	7	7	7	7	7	6	6	6	7	7	6	7	7	7	7	3	7	1	1	1	1	1	1	7	1	1	1	3	1	1	1	1	1	1	3	2	2	4
192	31	5941582	412	12	1	1	2	7	5	6	1	2	3	1				7	7	7	7	7	7	5	6	6	4	6	4	7	7	7	7	3	6	1	1	1	1	1	2	6	2	2	1	1	1	1	1	1	1	1	4	3	1	4
193	31	5941582	366	12	1	1	2	2	1	5	1	6	3	1				5	4	4	6	6	4	6	6	7	4	4	3	7	7	7	7	5	6	1	1	1	1	2	1	7	1	1	2	2	1	1	1	1	1	1	4	2	3	4
194	31	5941582	329	12	1	1	2	8	1	4	2	1	1	1				7												7	7	7	7	7	7	1	1	1	1	1	1	7	4	4	7	7	1	1							1	7
195	31	5941582	474	12	1	1	2	3	2	5	1	5	1	2				2	2	3	5	2	1	1	1	1	2	6	2	5	2	1	1	2	1	7	7	4	3	6	6	2	4	4	2	3	7	7	3	2	5	7	7	4	3	5
196	31	5941582	246	12	1	1	2	6	5	6	1	7	1	2				1	1	1	1	1	1	2	2	1	1	1	2	1	1	5	1	1	1	1	1	2	7	7	7	7	4	4	1	1	1	1	1	1	1	1	1	1	1	1
197	31	5941582	354	12	1	1	2	5	5	6	1	4	3	1				3	2	2	5	5	6	1	2	2	6	6	5	4	4	5	5	2	2	5	5	5	3	7	2	5	4	4	6	5	2	2	2	2	4	4	6	6	1	3
198	31	5941582	378	12	3	1	2	3	1	4	6	7	3	1				7	7	7	7	7	7	7	7	7	7	7	7	7	7	7	7	7	7	1	1	1	1	1	1	7	4	4	1	1	1	1	1	1	1	1	1	4	1	5
199	31	5941582	492	12	4	1	2	6	5	6	1	3	2	1				5	3	3	5	4	3	3	3	3	5	5	5	4	4	5	5	4	4	2	2	2	2	3	2	6	2	2	3	3	2	2	2	2	2	2	6	5	2	2
2	31	5941582	252	12	1	1	2	4	1	3	1	5	1	1				7	6	6	7	7	4	2	3	2	3	3	3	7	7	7	7	7	7	1	1	1	1	1	1	7	3	3	1	1	1	1	1	1	1	1	6	6	1	7
21	31	5941582	41	12	1	1	2	4	5	4	1	5	3	1				6	2	2	4	4	4	2	2	2	5	3	4	2	2	5	4	6	6	4	2	2	2	4	6	6	4	4	1	1	1	1	1	1	1	1	4	4	2	2
22	31	5941582	381	12	1	1	2	5	5	6	1	4	3	1				7	5	6	6	6	6	6	6	6	7	6	6	6	6	6	6	6	6	1	2	1	2	2	2	7	2	2	3	3	5	3	2	1	3	2	5	4	3	5
23	31	5941582	321	11	3	1	2	3	5	6	1	6	3	1				2	2	2	1	4	4	3	2	1	3	2	4	3	1	1	2	2	2	6	2	2	2	2	2	2	3	3	1	1	2	2	2	1	1	2	3	2	1	2
24	31	5941582	476	12	1	1	2		5	5	1	6	5	1				7	5	4	6	7	5	4	4	4	5	5	4	5	5	6	5	3	6	3	3	3	1	2	2	5	4	4	2	2	2	2	2	2	2	2	4	4	3	5
25	31	5941582	48	12	1	1	1	4	5	4	1	6	1	1	1	2	3	6	4	5	7	7	4	2	2	2	4	5	4	6	5	7	7	7	7	5	5	5	2	7	6	3	6	6	3	3	5	6	4	5	6	5	5	5	3	3
26	31	5941582	34	12	1	1	2	4	5	4	1	2	1	1				6	6	6	6	5	5	7	7	7	5	5	4	6	6	7	7	5	7	3	3	1	1	2	2	6	3	2	1	1	1	1	1	1	1	1	2	2	2	4

27	31	5941582	393	12	1	1	2	4	5	3	1	5	5	1				7	6	7	7	6	5	6	6	6	7	7	7	7	7	7	7	7	7	1	2	1	1	1	1	7	2	2	1	1	1	1	1	1	1	1	6	6	2	4
28	31	5941582	412	12	1	1	2	4	5	4	1	6	3	1				7	7	7	7	7	6	4	6	6	5	7	6	7	7	7	7	5	7	1	1	1	1	1	1	7	2	3	1	1	1	1	1	1	1	1	7	4	2	7
29	31	5941582	646	12	1	1	2	6	5	3	2	6	1	1				6	5		7	7		5	5	5		6	7	5	6	7	6	7	7	6	1	1	3	3	2	7	2	2	2	2	2	2	1	1	2	2	3	3	1	4
21	31	5941582	499	12	1	1	2	5	1	6	1	4	3	1				6	5	6	7	7	7	6	6	6	7	7	7	6	6	6	5	7	7	1	1	1	3	2	2	2	4	4	5	4	5	2	2	2	2	2	2	2	2	1
211	31	5941582	573	12	1	1	2	6	5	3	1	6	1	1				6	6	6	7	6	6	6	6	6	6	5	6	6	6	6	5	5	6	2	1	1	1	2	2	7	5	2	2	2	3	3	1	4	3	3	6	5	3	4
212	31	5941582	896	12	1	1	2	3	1	4	1	6	1	1				7	5	5	7	7	7	5	5	5	6	6	6	6	6	6	6	6	6	2	1	1	2	3	2	7	2	2	2	2	1	1	1	1	1	1	4	4	2	3
213	31	5941582	777	12	1	1	2	6	1	6	1	4	3	2				6	4	6	7	5	7	6	7	6	6	7	6	7	6	7	7	6	7	2	1	1	2	1	2	7	3	3	2	1	2	3	1	2	3	2	4	1	2	4
214	31	5941582	781	12	1	1	2	6	5	4	1	6	3	1				7	6	6	6	7	5	7	6	6	6	6	6	6	6	7	7	5	6	2	2	2	1	1	1	7	2	2	6	7	6	4	5	4	7	4	7	7	1	6
215	31	5941582	623	12	1	1	2	4	5	3	1	6	3	1				7	4	4	7	7	7	6	6	6	4	6	5	7	7	7	7	7	7	1	1	1	1	2	1	7	6	6	3	3	2	1	1	2	2	2	3	3	2	6
216	31	5941582	523	12	1	1	2	7	1	6	2	2	3	1				3	1	1	1	1	1	1	3	1	1	1	1	1	1	1	1	4	1	7	3	7	4	7	5	4	3	4	7	5	7	6	7	5	7	6	7	7	4	3
217	31	5941582	823	12	1	1	2	3	5	6	1	7	5	1				3	4	3	7	6	6	5	5	5	6	6	6	4	4	5	5	6	7	4	3	1	2	5	6	6	4	4	2	3	2	2	2	2	2	2	4	3	3	1
218	31	5941582	422	12	1	1	2	5	5	4	1	5	1	1				7	5	6	7	7	6	5	6	6	6	6	5	5	6	7	6	1	6	1	4	4	1	3	2	5	1	1	5	5	3	3	1	1	3	3	5	6	1	5
219	31	5941582	5	12	1	1	1	6	5	7	1	3	3	1	4	4	8	7	7	7	7	7	7	7	7	7	7	7	7	7	7	7	7	7	7	1	1	1	1	1	1	7	1	1	3	3	1	1	1	1	1	1	7	7	1	4
22	31	5941582	614	12	1	1	2	6	5	3	1	6	3	1				5	2	5	7	2	4	4	5	5	6	5	5	4	4	5	3	6	6	2	1	1	1	1	1	7	5	5	2	3	2	1	1	1	2	1	2	1	4	4
221	31	5941582	659	12	1	1	2	6	1	6	1	3	3	1				7	7	7	5	7	6	6	6	6	4	4	4	4	4	4	4	2	2	3	5	4	2	2	2	2	2	2	4	4	2	2	1	1	1	1	1	1	1	3
222	31	5941582	68	12	1	1	2	5	1	4	1	7	3	1				7	7	7	4	5	2	6	6	7	4	4	4	7	7	7	6	1	7	1	2	2	1	1	7	7	1	1	7	7	6	4	1	2	2	2	7	7	4	6
223	31	5941582	1164	12	1	1	2	6	5	4	1	6	1	1				7	7	1	7	7	7	1	1	1	1	4	6	1	1	7	4	4	4	1	1	1	1	7	7	4	4	4	1	1	1	1	1	1	1	1	1	1	1	1
224	31	5941582	495	12	1	1	2	4	5	6	1	4	3	1				7	7	7	7	7	7	7	7	7	7	7	7	7	7	7	7	1	7	1	1	1	1	1	1	7	1	1	5	5	1	1	1	1	2	2	7	7	1	4
225	31	5941582	542	12	1	1	2	5	1	5	1	6	1	1				1	1	1	4	1	1	1	1	1	4	4	3	1	1	5	5	1	7	1	1	1	1	7	7	7	1	5	1	4	1	1	1	1	1	4	1	1	1	1
226	31	5941582	88	12	1	1	1	5	5	4	1	6	3	2	1	29		4	3	3	6	6	6	5	4	5	7	6	7	7	7	7	7	7	7	2	2	1	1	2	1	7	2	2	6	5	3	3	2	2	3	3	5	3	3	3
227	31	5941582	345	12	1	1	2	3	2	7	1	5	3	1				1	1	1	1	1	1	1	4	4	4	4	4	1	1	1	1	1	4	1	3	7	4	4	4	4	4	4	4	4	4	4	5	4	4	4	4	4	5	4
228	31	5941582	36	12	3	1	2	5	5	4	1	5	3	1				6	1	1	1	1	1	1	1	1	1	1	1	1	1	1	1	1	1	1	1	5	6	7	7	1	7	7	4	4	7	5	2	7	4	7	7	7	1	1
229	31	5941582	618	12	1	1	1	5	5	4	3	6	3	1	1	33		4	3	3	6	5	5	6	6	5	3	4	5	4	4	4	5	7	7	3	2	1	5	4	4	5	4	4	1	2	3	4	1	2	1	3	4	3	4	5
23	31	5941582	333	12	1	1	2	4	5	3	1	6	3	1				7	6	7	7	7	7	7	7	7	7	7	7	7	7	7	7	6	6	1	1	1	1	1	1	7	2	2	1	1	1	1	1	1	1	1	7	4	1	7
231	31	5941582	818	12	1	1	2	6	5	3	1	4	5	1				7	7	7	7	7	7	6	7	7	7	7	7	7	7	7	7	7	7	1	1	1	1	1	1	7	1	1	6	6	1	1	1	1	1	1	6	7	1	2
232	31	5941582	535	12	1	1	2	7	5	7	1	5	3	1				7	7	7	7	7	7	7	7	6	7	6	6	7	7	7	7	5	5	2	7	1	1	1	1	7	2	1	3	3	1	1	1	1	1	3	7	7	3	3
233	31	5941582	318	12	1	1	2	5	5	6	1	6	5	1				7	7	7	7	5	5	4	4	4	7	4	7	5	5	7	7	4	4	1	1	1	1	1	6	1	1	1	1	1	1	1	1	1	1	1	1	3	1	5
234	31	5941582	1754	12	1	1	2	7	5	4	1	3	3	1				5	5	4	4	5	5	4	3	4	5	4	4	5	5	2	2	4	2	5	4	5	3	4	2	5	5	4	7	7	2	2	3	5	6	6	6	7	6	2
235	31	5941582	498	12	1	1	2	5	1	6	1	7	1	1				5	4	4	7	4	4	4	3	4	4	4	4	5	5	5	5	2	5	2	5	4	2	4	3	6	4	4	6	6	5	3	4	6	4	3	5	6	2	5
236	31	5941582	546	12	1	1	2	5	5	7	1	7	1	1				3	1	1	5	6	2	3	3	3	5	7	3	2	2	3	2	1	3	2	2	2	6	7	4	6	4	4	1	2	2	2	1	2	4	2	5	3	6	3

237	31	5941582	285	12	1	1	2	3	1	6	1	5	3	1				6	5	6	7	7	7	4	4	4	7	7	7	6	6	6	6	7	7	2	2	1	2	3	3	6	3	2	1	1	2	4	1	1	1	1	4	4	3	5
238	31	5941582	592	12	1	1	2	7	5	3	1	3	3	1				7	7	7	7	7	7	7	7	7	7	7	7	7	7	7	7	7	7	1	1	1	1	1	1	7	1	1	3	3	1	1	1	1	1	3	5	5	2	3
239	31	5941582	475	12	1	2	1	7	1	7	1	2	1	2	4	35	8	7	6	7	7	7	7	6	5	5	4	4	4	7	7	7	7	2	7	1	1	1	1	2	1	7	2	2	2	2	1	1	1	2	2	2	7	6	2	7
24	31	5941582	776	12	1	1	1	3	5	6	1	6	3	1	1	4		3	3	3	7	7	5	7	7	7	7	7	4	7	7	7	7	6	6	2	1	1	1	1	1	7	1	5	1	1	1	1	1	1	1	1	6	5	2	6
241	31	5941582	384	12	1	1	2	2	5	3	1	6	5	1				6	7	7	7	6	6	6	7	6	6	6	5	7	7	7	7	7	7	1	1	1	1	4	2	6	5	5	1	2	2	1	1	1	1	1	3	2	1	6
242	31	5941582	565	12	1	1	2	4	5	6	1	6	5	1				7	6	6	7	5	5	7	7	7	6	6	6	6	6	7	7	6	6	1	1	1	1	2	2	7	2	2	2	2	2	2	1	1	1	1	4	4	2	3
243	32	5941582	-1	12	1	1	2	4	1	6	1	6	3	1				7	6	6	7	5	5	6	6	6	4	6	5	6	6	7	7	5	7	2	2	2	2	2	1	6	2	2	2	3	2	2	1	2	1	2	5	5	3	5
244	31	5941582	67	12	4	1	2	4	1	6	1	6	3	1				2	1	2	2	1	1	3	3	3	2	2	2	1	1	4	1	7	4	6	1	1	1	7	7	7	1	1	4	4	1	1	1	1	1	1	1	1	1	2
245	31	5941582	433	12	1	1	2	3	5	6	1	6	3	1				7	6	6	7	6	6	3	3	3	6	6	6	7	6	6	5	4	4	6	5	5	2	3	4	3	4	4	1	1	5	4	2	1	1	4	5	5	2	2
246	31	5941582	59	12	1	1	1	6	1	6	1	6	3	1	3	15	9	6	6	7	7	7	6	6	6	6	7	7	7	7	7	6	6	5	6	1	1	1	2	2	7	7	2	2	3	3	1	1	1	1	1	1	4	3	1	6
247	31	5941582	162	12	3	1	2	5	5	8	1	6	1	1				1	4	1	4	4	4	4	4	4	2	3	1	1	1	4	4	1	1	7	7	7	1	7	3	1	3	3	5	4	3	3	3	4	4	3	7	7	3	3
248	31	5941582	392	12	1	1	1	6	5	4	2	6	1	1	1	18	18	6	6	6	6	6	7	2	1	2	3	3	3	5	5	6	6	7	4	1	5	1	2	2	5	5	3	2	3	4	2	2	4	6	4	4	4	4	2	4
249	31	5941582	8	12	1	1	2	6	1	3	1	3	3	1				3	3	2	3	5	2	3	3	3	4	3	3	1	1	4	3	7	7	3	3	1	2	2	2	6	3	3	1	1	1	1	1	1		1	2	2		2
25	31	5941582	654	12	1	1	1	5	5	4	2	6	3	1	1	6	17	7	7	7	7	7	7	5	5	4	7	6	7	7	7	7	7	5	7	2	1	1	1	2	2	7	1	1	1	1	1	1	1	1	1	1	4	2	1	7
251	31	5941582	477	12	1	2	2	7	1	6	1	3	3	1				7	7	6	7	7	7	5	5	5	7	6	7	7	7	6	6	7	7	5	7	1	1	1	1	7	1	1	3	1	1	1	1	1	1	1	6	7	1	7
252	31	5941582	613	12	1	1	2	6	1	6	1	3	1	2				7	4	4	7	7	7	7		7				7	7	7	7	7	7	1	1	1	1	1	2	6	2	3	1	5	1	1	1	1	1	1	3	1	1	6
253	31	5941582	577	12	1	1	2	4	1	6	1	6	3	1				6	6	7	7	4	4	6	6	6	6	7	5	6	5	6	6	3	4	1	3	1	1	1	2	6	3	3	1	1	5	4	1	2	3	1	5	3	1	2
254	31	5941582	551	12	1	1	2	6	5	4	1	4	3	1				7	7	7	7	7	7	7	7	7	7	7	7	7	7	7	7	7	7	1	7	1	1	1	1	7	1	1	4	5	2	2	1	1	1	1	7	7	2	2
255	31	5941582	791	12	1	1	2	6	5	4	1	3	3	2				7	7	7	7	7	6	6	7	7	6	6	6	7	7	7	6	7	7	6	2	1	1	1	1	7	1	1	6	5	6	4	4	4	4	4	4	5	4	4
256	31	5941582	358	12	1	1	1	6	1	4	1	3	3	2	3	45		7	6	5	6	6	6	7	6	6	6	6	6	6	6	5	6	6	6	1	1	1	1	1	1	7	2	2	2	2	1	1	1	1	2	2	4	3	3	2
257	31	5941582	1147	12	4	1	2	7	1	8	3	5	3	1				1	2	1	1	4	3	1	1	1	1	1	1	1	1	1	3	1	1	7	7	7	7	7	7	1	6	6	1	1	2	1	1	1	2	1	4	2	3	1
258	31	5941582	567	11	3	1	2	3	1	7	6	5	1	2				3	4	4	1		2	6	5	6	4	5	4	1	1	4	4	7	7	6	1	1	1	7	1	5	3	3	1	1	4	1	1	1	1	1	4	5	1	5
259	31	5941582	1288	12	1	1	2	6	1	6	1	5	1	1				6	6	6	5	6	5	1	1	2	5	5	5	6	6	7	5	7	7	1	1	1	1	3	2	7	1	2	1	1	1	1	1	1	1	1	4	2	1	2
26	31	5941582	452	12	1	1	2	6	1	6	1	3	3	1				2	2	2	2	6	6	6	6	6	3	3	3	5	5	5	6	6	6	2	2	2	2	6	2	6	1	1	1	1	1	1	1	1	1	1	6	6	1	1
261	31	5941582	8	12	1	1	1	5	5	6	1	6	1	1	3	36		7	7	7	6	7	6	6	6	6				7	7	6	4	5	6	1	1	1	1	1	2	7	3	4	3	3	1	1	1	1	2	1	4	4	2	
262	31	5941582	1181	12	1	1	2	7	5	3	1	3	3	2				7	7	7	7	5	5	7	7	7	7	7	7	7	7	7	7	3	7	1	1	1	1	1	1	7	5	2	3	5	1	1	1	1	1	1	1	1	1	1
263	31	5941582	514	12	1	1	2	2	5	3	1	6	3	1				5	5	3	6	6	2	4	3	4	2	2	2	5	5	5	2	6	6	3	1	1	2	2	3	6	7	7	2	2	2	2	2	6	2	3	5	4	3	5
264	31	5941582	584	12	1	1	2	3	5	6	1	6	5	1				6	5	5	6	6	6	6	6	6	7	7	4	6	6	5	5	5	5	1	1	1	1	7	5	7	2	2	1	1	1	1	1	1	1	1	3	2	3	5
265	32	5941582	-1	12	1	1	2	6	5	6	1	3	3	1				5	2	4	5	7	7	7	7	7	1	1	1	4	4	3	2	5	7	1	1	1	1	5	1	7	2	2	2	2	1	2	1	1	2	1	3	3	4	2
266	31	5941582	5	12	1	1	2	6	5	6	1	4	3	1				5	4	4	6	5	4	3	4	4	6	4	6	5	5	4	4	7	5	5	2	2	3	4	4	6	2	2	2	2	2	2	2	2	4	2	3	1	1	4

267	31	5941582	1742	12	5	1	1	6	1	4	5	6	4	2	3	44	8	7	6	7	7	6	6	7	6	6	6	6	5	7	6	7	7	7	7	1	2	1	2	2	2	7	3	4	5	6	5	6	4	6	5	2	6	7	4	3
268	31	5941582	457	12	1	1	2	6	1	6	1	3	3	1				7	7	7	6	7	7	6	7	7	4	4	3	7	7	7	6	7	7	1	1	1	1	1	1	7	2	2	1	1	1	1	1	1	1	1	6	6	2	6
269	31	5941582	145	12	1	1	2	6	1	9	3	5	2	1				7	7	7	7	7	7							7		7	7	4	4			4						4	4	4	1	4	1	1	4	4	7	7	1	1
27	32	5941582	-1	12	1	1	2	6	5	4	1	3	1	1				7	6	7	7	7	7	6	6	6	6	6	6	7	7	7	7	6	6	2	6	2	1	1	2	6	2	2	2	2	2	2	2	2	2	2	6	4	2	2
271	31	5941582	614	12	1	1	2	7	5	6	1	2	3	1				7	7	7	7	7	7	7	7	7	7	7	7	7	7	7	7	7	7	1	1	1	1	1	1	7	2	2	3	5	1	1	1	1	2	1	3	4	2	3
272	31	5941582	524	12	1	1	1	4	5	8	1	6	3	1	1	1		7	6	7	6	4	3	5	5	5	7	7	6	7	7	5	4	4	6	2	1	1	1	2	1	7	4	4	3	3	1	1	1	2	2	2	6	2	2	1
273	31	5941582	147	12	1	1	2	6	1	8	1	2	3	1				7	1	1	1	1	1	7	7	6	1	4	2	1	1	1	1	7				1	2	7	2	2		4	3	2	1	4	1	4	6	7	7	7	4	6
274	31	5941582	1573	12	1	1	2	6	5	9	1	3	3	1				3	3	3	2	4	3	1	1	1	3	3	3	3	3	3	3	3	3																					
275	31	5941582	165	12	1	1	2	5	1	4	1	5	3	2				6	6	6	4	5	5	4	4	5	4	4	4	6	6	7	6	3	4	4	5	3	1	1	1	7	4	4	5	5	5	6	2	2	6	2	6	5	2	5
276	31	5941582	3294	12	1	1	2	5	5	4	1	5	3	1				7	7	7	7	7	7	7	7	7	4	7	4	7	7	7	7	1	7	1	1	1	1	1	1	7	4	4	6	6	6	6	4	4	7	5	7	7	1	6
277	31	5941582	844	12	1	1	2	3	5	6	1	6	5	1				6	4	5	6	6	6	6	5	6	7	6	7	6	6	6	6	5	4	1	1	1	2	3	2	7	1	1	1	1	1	1	1	1	1	1	5	5	1	4
278	31	5941582	566	12	1	1	1	7	5	4	1	4	1	1	3	4		7	6	6	7	7	7	5	6	6	7	7	7	7	7	7		7	7	1	1	1	1	1	1	7	7	7	1	1	1	1	1	1	1	1	7	7	1	1
279	31	5941582	581	12	1	1	2		1	6	1	3	5	1				2	2	2	6	4	4	1	2	1	2	2	2	2	6	6	6	7	7	3	2	1	5	7	7	5	2	2	2	1	1	1	1	1	2	2	2	3	2	1
28	31	5941582	524	12	3	1	2	3	5	6	1	6	7	1				1	5	5	3	4	4	3	3	3	3	3	3	3	3	1	2	1	3	5	3	5	2	6	5	2	1	1	1	1	1	1	1	1	1	1	1	1	1	1
281	31	5941582	1483	12	1	1	2	5	5	6	1	4	3	1				7	7	7	7	7	7	7	7	7	7	7	7	7	7	7	7	7	7	1	1	1	1	1	1	7	1	1	1	1	1	1	1	2	1	1	2	2	1	1
282	31	5941582	123	12	1	1	2	7	1	7	5	2	3	1				7	7	7	7	7	7	7	7		7	7	7	7	7	7	7	5	4	1	7	1									1	1	1	1	2	2	7	7	1	7
283	31	5941582	311	12	1	1	2	3	5	6	1	7	3	1				7	5	6	6	4	5	6	6	6	5	5	4	7	7	7	6	5	5	3	3	2	2	1	1	6	4	4	2	2	7	5	2	5	4	6	7	7	4	2
284	31	5941582	1329	12	1	1	2	7	1	6	1	2	3	1				6	1	1	7	7	6	5	5	5				1	1	3	3	1	1	1	4	5	1	7	2	4	1	1	3	3	1	1	1	1	1	6	5	5	1	1
285	31	5941582	169	12	1	6	2	6	5	3	5	4	3	1				7	1	1	7	1	7	1	1	1	4	4	4	1	1	2	2	1	1	7	2	4	7	7	7	4	1	1	4	7	1	1	3	4	4	4	7	7	2	4
286	31	5941582	1342	12	1	1	2	7	1	4	4	2	3	1				7	7	7	7	5	7	4	5	5	4	4	3	7	5	6	5	5	5	3	4	4	5	7	5	4	4	4	5	6	2	2	5	2	3	3	7	7	4	3
287	31	5941582	518	12	1	1	2	4	5	7	1	7	1	1				7	6	7	7	7	6	7	7	7	7	7	7	7	7	5	4	4	6	1	1	1	1	1	1	7	2	2	2	2	2	3	1	1	2	1	5	5	2	2
288	31	5941582	455	12	1	1	2	2	5	5	1	5	3	1				2	2	3	1	2	3	6	5	6	5	5	5	5	6	5	4	7	7	2	2	1	2	4	3	7	1	1	2	1	1	1	1	1	1	2	3	4	2	5
289	31	5941582	48	12	1	1	2	3	5	6	1	4	5	1				7	4	6	4	5	5	3	3	3	5	5	5	4	4	4	3	7	7	1	2	1	2	2	5	6	3	3	2	2	2	2	1	1	2	2	3	3	3	3
29	31	5941582	842	12	1	1	2	5	5	6	1	6	1	3				5	4	7	7	6	6	7	7	7				7	7	7	7	3	4	2	3	1	1	1	1	7			3	4	4	4	2	3	2	2	6	5	1	5
291	31	5941582	39	12	1	1	2	5	5	6	1	6	6	1				4	2	2	5	4	4	3	1	3	5	5	4	2	3	7	5	2	3	4	6	3	2	3	3	5	4	4	5	5	2	2	2	2	2	2	4	3	2	2
292	31	5941582	141	12	1	1	2	6	5	4	1	5	3	1				7	4	4	7	7	7	3	3	3	5	5	5	6	6	7	7	5	6	1	1	1	3	3	2	6	2	4	5	3	1	2	2	5	3	2	5	6	2	4
293	31	5941582	712	12	1	1	2	6	5	6	1	4	3	1				7				7	7	5	5	5	6	6	6	7	6	5	4	7	7	2	2	1	1	1	2	6	4	4	1	1	1	1	1	1	6	2	4	4	2	2
294	31	5941582	466	12	1	1	2	5	5	6	1	6	3	1				5	3	6	7	4	7	6	6	6	6	6	6	6	6	6	4	4	4	1	2	1	1	1	1	7	4	4	3	3	4	2	2	2	2	2	6	4	3	3
295	31	5941582	449	12	1	1	2	5	5	4	1	4	3	1				5	3	5	7	5	5	1	1	1	3	3	1	5	3	7	7	7	7	1	1	1	1	7	1	7	1	1	1	1	7	1	1	1	1	1	1	7	1	3
296	31	5941582	493	12	1	1	2	2	5	3	3	7	2	1				3	6	5	4	2	1	7	7	7	4	5	6	6	7	7	5	5	7	1	1	1	2	2	2	6	4	3	1	1	2	2	1	1	1	1	5	2	3	6

297	31	5941582	747	12	1	1	2	6	5	3	1	3	3	1				7	7	7	7	7	7	5	5	5	6	6	7	7	7	7	7	7	7	1	1	1	1	1	1	7	1	1	1	1	1	1	1	1	1	1	3	3	1	1
298	31	5941582	341	12	1	1	2	4	5	6	1	6	3	1				5	5	5	7	6	5	6	6	4	7	7	7	7	7	7	5	6	6	1	1	1	1	1	1	7	6	6	3	4	3	1	1	1	3	2	5	5	3	1
299	31	5941582	229	12	1	1	2	6	5	8	1	5	1	1				7	7	7	2	7	2	4	7	6	5	5	4	4	4	5	7	1	1	7	7	7	4	1	7	5	1	1	7	7	7	7	4	7	7	7	7	7	3	4
3	31	5941582	969	12	1	1	2	5	5	3	1	7	1	1				1	3	4	7	7	6	7	7	7	7	4	2	3	3	6	3	3	3	4	4	3	7	7	4	5	5	5	2	4	4	3	1	2	2	4	5	4	3	5
31	31	5941582	416	12	1	1	2	6	5	3	1	6	1	1				7	5	3	7	3	3	2	3	2	4	3	3	3	5	5	3	5	5	1	2	1	3	5	5	6	2	2	5	6	2	2	2	1	3	3	7	3	2	3
32	31	5941582	1898	12	1	1	2	6	5	3	1	3	3	2				7	4	4	7	4	4	6	6	6	7	7	7	7	7	7	7	7	7	1	1	1	1	1	1	7	7	1	2	4	1	1	1	1	3	3	4	4	1	4
33	31	5941582	656	12	1	1	2	6	5	4	1	6	6	1				7	2	6	7	6	6	7		6	7			6	6	5	5	6	6	1	1	1	2	3	2		4	4	5	7	1	1	1	4	5	5	5	6	1	1
34	31	5941582	526	12	4	1	2	2	5	3	1	6	6	2				4	1	4	1	1	1	1	1	1	1	1	1	1	1	1	1	7	6	1	1	1	4	7	7	4	2	1	1	1	1	1	1	1	2	1	1	1	1	1
35	31	5941582	651	12	1	1	1	3	5	5	1	6	3	1	1	4		7	3	6	3	7	5	7	3	7	3	3	3	7	7	7	4	7	5	1	2	1	1	1	1	6	1	1	1	1	1	1	1	1	1	2	6	6	1	7
36	31	5941582	1413	12	1	1	2	6	5	4	1	3	3	1				7	7	7	7	7	7	7	7	7	7	7	7	7	7	7	5	4	7	1	1	1	1	1	1	7	5	5	7	7	2	2	1	1	2	1	7	4	1	3
37	31	5941582	412	11	1	1	2	5	5	6	1	5	1	1				4	1	3	2	5	4	7	7	7	1	1	1	2	2	1	5	6	4	1	2	1	1	4	1	7	1	1	1	2	1	1	1	1	1	1	2	4	1	1
38	31	5941582	466	12	1	1	2	6	5	3	1	3	3	1				7	6	6	7	6	6	5	5	5	6	6	7	7	7	7	7	7	4	2	1	4	1	1	1	7	4	6	6	6	7	7	2	6	7	5	7	7	5	6
39	31	5941582	157	12	1	1	2	6	5	6	1	4	3	1				3	3	3	2	2	2	3	3	3	4	4	4	3	4	2	3	5	2	6	5	4	5	7	4	5	3	4	2	2	1	1	1	1	1	2	6	3	1	1
31	31	5941582	595	12	1	1	1	6	5	6	1	6	3	1	3	35	1	7	7	7	7	7	7	7	7	7	7	7	7	7	7	7	7		7	1	1	1	1	1	1	7	1	1	4	4	1	1	1	1	1	1	5	5	1	7
311	31	5941582	869	12	1	1	2	6	5	3	1	3	1	1				5	5	5	7	7	6	5	2	4	4	2	4	5	6	7	7	5	5	1	1	1	1	1	6	7	6	7	5	7	1	1	1	1	1	1	3	2	1	3
312	31	5941582	46	12	1	1	2		5	3	1	5	3	1				7	6	7	7	7	3	4	4	5	7	5	5	7	7	7	6	7	7	6	1	1	1	1	2	7	3	3	1	7	1	1	1	1	1	1	7	7	1	4
313	31	5941582	645	12	3	1	2	6	5	5	6	4	3	2				2	1	1	1	1	1	4	4	5	2	2	3	1	1	2	3	1	1	7	6	2	1	7	2	6	4	4	2	2	2	2	2	4	2	2	4	4	3	2
314	32	5941582	-1	12	3	1	2	4	5	6	1	5	3	1				7	5	5	5	6	6	6	5	6	7	7	7	7	7	7	6	6	6	1	1	1	1	1	1	7	2	2	1	1	1	1	1	1	1	1	2	1	1	1
315	31	5941582	216	12	1	1	2	2	1	3	1	4	3	1				6	6	5	6	4	4	6	5	5	4	5	4	5	5	6	4	4	4	2	1	3	2	2	2	6	2	2	2	3	1	2	1	2	2	1	2	2	2	4
316	31	5941582	327	12	1	1	2	7	1	4	1	3	3	2				6	5	6	6	6	6	5	3	5	6	5	5	6	6	5	5	5	5	1	1	1	2		2	7	4	4	5	5	1	1	3	3	3	2	4	3	4	4
317	31	5941582	943	12	1	1	2	6	1	4	1	4	1	1				7	6	7	7	7	7	7		7	6	6		7	7	7	7	7	7	1	7	1	1	1	1	7	5	5	5	6	7	3	4	5	5	5	7	7	4	3
318	31	5941582	765	12	1	1	2	3	5	6	1	5	3	1				6	5	5	7	7	7	7	7	7	1	1	1	6	6	7		6	7	1	1	1	1	1	2	7	4	5	3	2	1	1	1	1	2	1	6	3	3	5
319	31	5941582	716	12	1	1	2	5	5	7	1	6	3	1				7	7	7	7	7	5	6	6	7	7	7	7	7	7	7	7	7	7	1	1	1	2	2	1	7	4	4	3	3	2	1	1	1	1	1	1	1	1	1
32	31	5941582	726	12	1	4	2	4	1	4	6	1	7	1				7	5	5	4	5	5	5	5	5	5	3	3	5	5	5	4	5	5	1	1	1	1	3	2	6	2	2	2	2	2	2	1	1	2	2	6	5	2	2
321	31	5941582	57	12	1	1	2	1	5	2	6	8	7	3				7	4	1	7	7	6	5	7	7	7	7	7	7	7	7	4	7	7	1	1	1	1	1	1	7	1	1	1	1	1	1	1	1	1	1	5	2	3	1
322	31	5941582	438	12	1	1	2	5	1	6	1	4	1	1				6	4	7	7	7	4	1	1	1	6	4	4	5	5	6	5	6	6	1	1	1	1	1	3	7	4	2	1	4	1	1	1	1	1	1	5	4	1	4
323	31	5941582	141	12	1	1	2	2	5	3	1	7	1	2				7	6	7	6	6	6	7	7	7	4	4	4	6	7	7	7	4	7	1	1	1	1	1	1	7	1	1	1	2	1	1	1	1	1	1	1	4	1	7
324	31	5941582	928	12	1	1	2	5	5	6	1	4	3	1				7	1	7	7	4	6	7	7	7	7			7	7	7	7	5	5	1	5	1	1	1	1	7	2	2	4	4	4	3	3	4	4	3	5	3	5	6
325	31	5941582	64	12	1	1	2	4	5	4	1	6	3	1				6	3	3	3	5	5	5	5	5	5	6	6	6	5	6	6	4	4	2	2	2	3	3	2	6	5	5	5	5	4	2	3	5	5	3	6	6	2	4
326	31	5941582	37	12	1	1	2	4	5	7	1	5	3	1				5	5	5	6	4	3	5	5	5	1	1	1	5	6	5	3	6	6	2	4	2	3	5	3	6	5	5	3	3	2	2	1	1	4	4	2	2	2	1

327	31	5941582	67	12	1	1	2	3	1	4	1	7	3	1				7	7	7	7	7	7	7	7	7	3	3	3	7	7	7	7	4	4	1	1	1	1	1	1	7	4	4	4	4	7	4	1	1	1	1	4	5	1	4
328	31	5941582	584	12	1	1	2	3	5	6	1	6	5	1				6	6	6	6	6	7	6	6	6	6	6	6	5	5	7	7	6	6	1	1	1	1	2	1	7	2	2	4	4	1	1	2	2	2	3	4	4	2	2
329	31	5941582	396	12	1	1	1	3	1	4	1	6	1	1	2	1	6	7	5	6	7	6	6	6	4	5	5	5	6	6	4	4	4	5	5	3	6	5	5	3	3	3	3													
33	31	5941582	34	12	1	1	2	1	1	3	1	8	1	1				7	6	2	7	2	2	6	6	7	5	1	1	6	7	7	5	2	7	1	1	1	2	1	2	2	1	1	1	1	1	1	1	1	1	1	4	5	2	1
331	31	5941582	518	12	1	1	2	4	1	5	1	5	3	1				6	7	6	7	7	7	7	7	7	4	4	4	7	7	7	7	7	7	1	1	1	1	1	2	7	1	1	2	2	1	1	1	1	1	2	4	3	2	1
332	31	5941582	596	12	1	1	2	6	1	4	1	5	3	1				2	2	4	7	4	2	4	3	4	1	2	4	6	4	1	1	4	4	1	4	4	4	3	4	4	1	1	3	3	1	5	1	1	1	4	2	2	1	4
333	31	5941582	785	12	1	1	1	5	1	6	1	6	5	1	1	7		2	1	7	1	1	1	1	1	1	1	5	5	1	1	1	1	2	2	7	7	7	7	7	3	2	5	5	7	7	1	7	7	7	7	7	7	2	7	7
334	31	5941582	1333	12	1	1	2	2	5	3	1	7	1	1				6	7	7	7	7	7	6	5	6	6	7	4	7	7	7	6	7	7		2	4	1	2	1	7	4	4	5	4	5	7	7	7	7	3	6	2	3	6
335	32	5941582	-1	12	1	1	2	5	5	6	1	5	1	1				6	6	5	5	7	6	5	5	5	7	7	7	6	6	7	7	7	7	1	1	1	1	2	1	7	2	2	1	1	1	1	1	1	1	1	6	3	1	7
336	31	5941582	41	12	1	1	2	2	1	3	1	5	3	1				7	5	3	7	6	7	7	5	6	4	6	5	6	6	7	7	4	4	2	2	1	2	2	2	7	6	3	1	2	5	1	1	1	2	5	7	6	2	6
337	31	5941582	125	12	1	1	1	2	5	3	1	1	3	2	1	2		7	7	7	7	6	5	4	3	3	6	6	6	7	7	7	5	7	6	1	1	1	1	1	1	7	3	4	1	2	1	1	1	1	1	1	1	1	1	1
338	31	5941582	54	12	1	1	2	6	5	3	2	2	3	1				4	4	4	4	6	4	4	4	4	4	4	4	6	5	6	4	3	2	4	4	7	4	4	4	4	4	4	5	6	4	3	2	3	4	3	4	5	4	4
339	31	5941582	6	12	1	1	2	2	5	3	1	7	3	1				7	3	5	6	3	6	3	2	2	6	5	4	7	7	7	4	7	7	1	2	2	1	5	2	7	3	3	1	1	1	1	1	1	1	2	1	3	1	1
34	31	5941582	279	12	1	1	2	2	5	3	1	6	1	1				7	7	7	7	6	7	7	6	5	4	4	6	7	7	7	7	1	7	1	1	1	1	1	1	7	4	4	2	2	4	4	1	1	2	1	6	5	4	4
341	31	5941582	543	12	1	1	2	2	1	3	1	6	3	1				7	5	4	3	2	2	6	6	6	5	5	6	5	5	5	5	7	7	1	1	1	1	3	1	7	5	5	1	1	1	3	1	1	1	1	4	3	1	1
342	31	5941582	588	12	1	1	1	3	5	4	1	6	3	1	1	1	5	6	7	7	7	7	5	7	7	7	7	7	7	7	7	7	5	7	7	1	1	1	2	1	1	7	6	2	1	1	1	1	2	2	2	2	7	1	1	7
343	31	5941582	636	12	1	6	2	3	5	3	2	7	1	2				7	7	7	7	7	7	7	7	7	7	7	5	7	7	7	7	7	7	1	1	1	1	1	5	7	3	2	1	1	1	1	1	1	1	1	7	7	4	5
344	31	5941582	178	12	1	1	2	6	5	6	1	4	3	1				4	2	2	6	3	5	6	6	6	6	4	5	6	5	6	6	6	6	2	2	2			6	6	5	5	4	3	3	3	2	2	2	2	5	6	3	3
345	32	5941582	-1	12	1	1	2	7	5	4	1	3	1	1				7	7	7	7	7	7	7	7	7	7	7	7	7	7	7	7	7	7	7	7	7	1	1	1	1	4	4	6	6	1	1	1	4	1	1	7	4	1	7
346	31	5941582	859	12	1	1	2	4	5	5	1	5	1	1				7	7	7	7	7	7	7	6	6	7	7	7	7	7	7	7	4	5	1	1	2	1	1	1	7	3	3	4	5	3	3	5	7	6	6	7	7	5	7
347	31	5941582	1354	12	1	1	2	5	1	6	1	6	5	1				2	2	1	6	5	6	3	3	3	1	1	1	1	1	2	1	6	6	6	4	4	2	6	7	5	4	4	2	2	2	2	2	2	3	2	4	4	2	2
348	31	5941582	392	12	1	1	2	3	1	6	1	6	3	1				2	6	7	5	4	5	7	7	7	7	7	1	4	3	2	2	7	7	1	1	1	1	1	1	7	5	6	3	2	2	1	1	1	1	1	5	5	1	1
349	31	5941582	58	12	1	1	2	5	1	3	1	7	1	1				6	7	6	7	7	7	6	6	6	6	6	6	6	6	7	7	6	6	1	1	1	2	2	2	7	2	2	2	2	2	2	1	1	2	2	7	7	2	2
35	32	5941582	-1	12	1	1	2	8	1	2	2	1	3	1				7	6	5	6	7	7	5	6	6	4	7	4	7	7	7	7	6	6	2	1	1	2	1	1	7	4	4	5	5	2	4	3	2	4	2	5	5	3	5
351	31	5941582	342	12	1	1	2	5	1	5	1	7	6	1				2	2	2	2	2	3	3	3	3	2	3	2	3	3	3	2	5	6	4	3	4	6	7	5	4	5	5	2	2	3	3	2	4	2	3	4	4	3	3
352	31	5941582	546	12	1	1	2	5	1	3	1	5	1	1				7	7	7	7	7	7	5	5	5	6	7	7	7	7	7	7	7	7	1	1	1	1	2	1	7	1	1	1	1	1	1	1	1	1	1	1	1	1	1
353	31	5941582	1483			1	1		5	2	2	5	2		1	5	4	1	1	1	1	1	1				1	1	1	1	1	1	1	7	1	1	1	1	7	7	7	7	1	1	7	7	1	1	1	1	1	2	4	6	3	3
354	31	5941582	1515			1	1		5	1	1	1	1		1	8	8	1	1	1	1	1	1	1	1	1	1	1	1	1	1	1	1	1	1	1	1	1	7	7	7	7	1	1	7	7	1	1	1	1	1	1	1	1	1	1
355	31	5941582	1458			3	1		1	6	1	6	4		1	16	5	4	4	4	4	4	4	4	4	4	4	4	4	4	4	4	4	4	4	4	4	4	4	4	4	4	4	4	4	4	4	4	4	4	4	4	4	4	4	4
356	31	5941582	54			1	1		1	6	1	6	1		1	3	6	4	4	4	4	4	4	4	4	4	4	4	4	4	4	4	4	4	4	4	4	4	4	4	4	4	4	4	4	4	4	4	4	4	4	4	4	4	4	4

357	31	5941582	214			1	1		5	3	2	4	1		1	78	12	6	5	3	5	4	4	4	4	4	6	4	5	5	5	5	5	5	5	5	5	5	3	3	3	3	5	5	2	2	4	4	4	4	4	4	5	5	5	5
358	31	5941582	47	12	1	1	1	4	5	4	1	6	1	1	3	26	7	7	7	7	7	7	7	6	6	6	7	7	6	7	7	7	7	3	7	1	1	1	1	1	1	7	1	1	1	3	1	1	1	1	1	1	3	2	2	4
359	31	5941582	48	12	1	1	1	4	5	4	1	6	1	1	1	2	3	6	4	5	7	7	4	2	2	2	4	5	4	6	5	7	7	7	7	5	5	5	2	7	6	3	6	6	3	3	5	6	4	5	6	5	5	5	3	3
36	31	5941582	5	12	1	1	1	6	5	7	1	3	3	1	4	4	8	7	7	7	7	7	7	7	7	7	7	7	7	7	7	7	7	7	7	1	1	1	1	1	1	7	1	1	3	3	1	1	1	1	1	1	7	7	1	4
361	31	5941582	88	12	1	1	1	5	5	4	1	6	3	2	1	29		4	3	3	6	6	6	5	4	5	7	6	7	7	7	7	7	7	7	2	2	1	1	2	1	7	2	2	6	5	3	3	2	2	3	3	5	3	3	3
362	31	5941582	618	12	1	1	1	5	5	4	3	6	3	1	1	33		4	3	3	6	5	5	6	6	5	3	4	5	4	4	4	5	7	7	3	2	1	5	4	4	5	4	4	1	2	3	4	1	2	1	3	4	3	4	5
363	31	5941582	475	12	1	2	1	7	1	7	1	2	1	2	4	35	8	7	6	7	7	7	7	6	5	5	4	4	4	7	7	7	7	2	7	1	1	1	1	2	1	7	2	2	2	2	1	1	1	2	2	2	7	6	2	7
364	31	5941582	776	12	1	1	1	3	5	6	1	6	3	1	1	4		3	3	3	7	7	5	7	7	7	7	7	4	7	7	7	7	6	6	2	1	1	1	1	1	7	1	5	1	1	1	1	1	1	1	1	6	5	2	6
365	31	5941582	59	12	1	1	1	6	1	6	1	6	3	1	3	15	9	6	6	7	7	7	6	6	6	6	7	7	7	7	7	6	6	5	6	1	1	1	2	2	7	7	2	2	3	3	1	1	1	1	1	1	4	3	1	6
366	31	5941582	392	12	1	1	1	6	5	4	2	6	1	1	1	18	18	6	6	6	6	6	7	2	1	2	3	3	3	5	5	6	6	7	4	1	5	1	2	2	5	5	3	2	3	4	2	2	4	6	4	4	4	4	2	4
367	31	5941582	654	12	1	1	1	5	5	4	2	6	3	1	1	6	17	7	7	7	7	7	7	5	5	4	7	6	7	7	7	7	7	5	7	2	1	1	1	2	2	7	1	1	1	1	1	1	1	1	1	1	4	2	1	7
368	31	5941582	358	12	1	1	1	6	1	4	1	3	3	2	3	45		7	6	5	6	6	6	7	6	6	6	6	6	6	6	5	6	6	6	1	1	1	1	1	1	7	2	2	2	2	1	1	1	1	2	2	4	3	3	2
369	31	5941582	8	12	1	1	1	5	5	6	1	6	1	1	3	36		7	7	7	6	7	6	6	6	6				7	7	6	4	5	6	1	1	1	1	1	2	7	3	4	3	3	1	1	1	1	2	1	4	4	2	
37	31	5941582	1742	12	5	1	1	6	1	4	5	6	4	2	3	44	8	7	6	7	7	6	6	7	6	6	6	6	5	7	6	7	7	7	7	1	2	1	2	2	2	7	3	4	5	6	5	6	4	6	5	2	6	7	4	3
371	31	5941582	524	12	1	1	1	4	5	8	1	6	3	1	1	1		7	6	7	6	4	3	5	5	5	7	7	6	7	7	5	4	4	6	2	1	1	1	2	1	7	4	4	3	3	1	1	1	2	2	2	6	2	2	1
372	31	5941582	566	12	1	1	1	7	5	4	1	4	1	1	3	4		7	6	6	7	7	7	5	6	6	7	7	7	7	7	7		7	7	1	1	1	1	1	1	7	7	7	1	1	1	1	1	1	1	1	7	7	1	1
373	31	5941582	651	12	1	1	1	3	5	5	1	6	3	1	1	4		7	3	6	3	7	5	7	3	7	3	3	3	7	7	7	4	7	5	1	2	1	1	1	1	6	1	1	1	1	1	1	1	1	1	2	6	6	1	7
374	31	5941582	595	12	1	1	1	6	5	6	1	6	3	1	3	35	1	7	7	7	7	7	7	7	7	7	7	7	7	7	7	7	7		7	1	1	1	1	1	1	7	1	1	4	4	1	1	1	1	1	1	5	5	1	7
375	31	5941582	396	12	1	1	1	3	1	4	1	6	1	1	2	1	6	7	5	6	7	6	6	6	4	5	5	5	6	6	4	4	4	5	5	3	6	5	5	3	3	3	3													
376	31	5941582	785	12	1	1	1	5	1	6	1	6	5	1	1	7		2	1	7	1	1	1	1	1	1	1	5	5	1	1	1	1	2	2	7	7	7	7	7	3	2	5	5	7	7	1	7	7	7	7	7	7	2	7	7
377	31	5941582	125	12	1	1	1	2	5	3	1	1	3	2	1	2		7	7	7	7	6	5	4	3	3	6	6	6	7	7	7	5	7	6	1	1	1	1	1	1	7	3	4	1	2	1	1	1	1	1	1	1	1	1	1
378	31	5941582	588	12	1	1	1	3	5	4	1	6	3	1	1	1	5	6	7	7	7	7	5	7	7	7	7	7	7	7	7	7	5	7	7	1	1	1	2	1	1	7	6	2	1	1	1	1	2	2	2	2	7	1	1	7
379	31	5941582	1483			1	1		5	2	2	5	2		1	5	4	1	1	1	1	1	1				1	1	1	1	1	1	1	7	1	1	1	1	7	7	7	7	1	1	7	7	1	1	1	1	1	2	4	6	3	3
38	31	5941582	1515			1	1		5	1	1	1	1		1	8	8	1	1	1	1	1	1	1	1	1	1	1	1	1	1	1	1	1	1	1	1	1	7	7	7	7	1	1	7	7	1	1	1	1	1	1	1	1	1	1
381	31	5941582	1458			3	1		1	6	1	6	4		1	16	5	4	4	4	4	4	4	4	4	4	4	4	4	4	4	4	4	4	4	4	4	4	4	4	4	4	4	4	4	4	4	4	4	4	4	4	4	4	4	4
382	31	5941582	54			1	1		1	6	1	6	1		1	3	6	4	4	4	4	4	4	4	4	4	4	4	4	4	4	4	4	4	4	4	4	4	4	4	4	4	4	4	4	4	4	4	4	4	4	4	4	4	4	4
383	31	5941582	214			1	1		5	3	2	4	1		1	78	12	6	5	3	5	4	4	4	4	4	6	4	5	5	5	5	5	5	5	5	5	5	3	3	3	3	5	5	2	2	4	4	4	4	4	4	5	5	5	5
384	31	5941582	47	12	1	1	1	4	5	4	1	6	1	1	3	26	7	7	7	7	7	7	7	6	6	6	7	7	6	7	7	7	7	3	7	1	1	1	1	1	1	7	1	1	1	3	1	1	1	1	1	1	3	2	2	4
385	31	5941582	48	12	1	1	1	4	5	4	1	6	1	1	1	2	3	6	4	5	7	7	4	2	2	2	4	5	4	6	5	7	7	7	7	5	5	5	2	7	6	3	6	6	3	3	5	6	4	5	6	5	5	5	3	3
386	31	5941582	5	12	1	1	1	6	5	7	1	3	3	1	4	4	8	7	7	7	7	7	7	7	7	7	7	7	7	7	7	7	7	7	7	1	1	1	1	1	1	7	1	1	3	3	1	1	1	1	1	1	7	7	1	4

387	31	5941582	88	12	1	1	1	5	5	4	1	6	3	2	1	29		4	3	3	6	6	6	5	4	5	7	6	7	7	7	7	7	7	7	2	2	1	1	2	1	7	2	2	6	5	3	3	2	2	3	3	5	3	3	3
388	31	5941582	618	12	1	1	1	5	5	4	3	6	3	1	1	33		4	3	3	6	5	5	6	6	5	3	4	5	4	4	4	5	7	7	3	2	1	5	4	4	5	4	4	1	2	3	4	1	2	1	3	4	3	4	5
389	31	5941582	475	12	1	2	1	7	1	7	1	2	1	2	4	35	8	7	6	7	7	7	7	6	5	5	4	4	4	7	7	7	7	2	7	1	1	1	1	2	1	7	2	2	2	2	1	1	1	2	2	2	7	6	2	7
39	31	5941582	776	12	1	1	1	3	5	6	1	6	3	1	1	4		3	3	3	7	7	5	7	7	7	7	7	4	7	7	7	7	6	6	2	1	1	1	1	1	7	1	5	1	1	1	1	1	1	1	1	6	5	2	6
391	31	5941582	59	12	1	1	1	6	1	6	1	6	3	1	3	15	9	6	6	7	7	7	6	6	6	6	7	7	7	7	7	6	6	5	6	1	1	1	2	2	7	7	2	2	3	3	1	1	1	1	1	1	4	3	1	6
392	31	5941582	392	12	1	1	1	6	5	4	2	6	1	1	1	18	18	6	6	6	6	6	7	2	1	2	3	3	3	5	5	6	6	7	4	1	5	1	2	2	5	5	3	2	3	4	2	2	4	6	4	4	4	4	2	4
393	31	5941582	654	12	1	1	1	5	5	4	2	6	3	1	1	6	17	7	7	7	7	7	7	5	5	4	7	6	7	7	7	7	7	5	7	2	1	1	1	2	2	7	1	1	1	1	1	1	1	1	1	1	4	2	1	7
394	31	5941582	358	12	1	1	1	6	1	4	1	3	3	2	3	45		7	6	5	6	6	6	7	6	6	6	6	6	6	6	5	6	6	6	1	1	1	1	1	1	7	2	2	2	2	1	1	1	1	2	2	4	3	3	2
395	31	5941582	8	12	1	1	1	5	5	6	1	6	1	1	3	36		7	7	7	6	7	6	6	6	6				7	7	6	4	5	6	1	1	1	1	1	2	7	3	4	3	3	1	1	1	1	2	1	4	4	2	
396	31	5941582	1742	12	5	1	1	6	1	4	5	6	4	2	3	44	8	7	6	7	7	6	6	7	6	6	6	6	5	7	6	7	7	7	7	1	2	1	2	2	2	7	3	4	5	6	5	6	4	6	5	2	6	7	4	3
397	31	5941582	524	12	1	1	1	4	5	8	1	6	3	1	1	1		7	6	7	6	4	3	5	5	5	7	7	6	7	7	5	4	4	6	2	1	1	1	2	1	7	4	4	3	3	1	1	1	2	2	2	6	2	2	1
398	31	5941582	566	12	1	1	1	7	5	4	1	4	1	1	3	4		7	6	6	7	7	7	5	6	6	7	7	7	7	7	7		7	7	1	1	1	1	1	1	7	7	7	1	1	1	1	1	1	1	1	7	7	1	1
399	31	5941582	651	12	1	1	1	3	5	5	1	6	3	1	1	4		7	3	6	3	7	5	7	3	7	3	3	3	7	7	7	4	7	5	1	2	1	1	1	1	6	1	1	1	1	1	1	1	1	1	2	6	6	1	7
4	31	5941582	595	12	1	1	1	6	5	6	1	6	3	1	3	35	1	7	7	7	7	7	7	7	7	7	7	7	7	7	7	7			7	1	1	1	1	1	1	7	1	1	4	4	1	1	1	1	1	1	5	5	1	7
41	31	5941582	396	12	1	1	1	3	1	4	1	6	1	1	2	1	6	7	5	6	7	6	6	6	4	5	5	5	6	6	4	4	4	5	5	3	6	5	5	3	3	3	3													
42	31	5941582	785	12	1	1	1	5	1	6	1	6	5	1	1	7		2	1	7	1	1	1	1	1	1	1	5	5	1	1	1	1	2	2	7	7	7	7	7	3	2	5	5	7	7	1	7	7	7	7	7	7	2	7	7
43	31	5941582	125	12	1	1	1	2	5	3	1	1	3	2	1	2		7	7	7	7	6	5	4	3	3	6	6	6	7	7	7	5	7	6	1	1	1	1	1	1	7	3	4	1	2	1	1	1	1	1	1	1	1	1	1
44	31	5941582	588	12	1	1	1	3	5	4	1	6	3	1	1	1	5	6	7	7	7	7	5	7	7	7	7	7	7	7	7	7	5	7	7	1	1	1	2	1	1	7	6	2	1	1	1	1	2	2	2	2	7	1	1	7
45	31	5941582	651	12	1	1	1	3	5	5	1	6	3	1	1	4		7	3	6	3	7	5	7	3	7	3	3	3	7	7	7	4	7	5	1	2	1	1	1	1	6	1	1	1	1	1	1	1	1	1	2	6	6	1	7
46	31	5941582	595	12	1	1	1	6	5	6	1	6	3	1	3	35	1	7	7	7	7	7	7	7	7	7	7	7	7	7	7	7			7	1	1	1	1	1	1	7	1	1	4	4	1	1	1	1	1	1	5	5	1	7
47	31	5941582	392	12	1	1	1	6	5	4	2	6	1	1	1	18	18	6	6	6	6	6	7	2	1	2	3	3	3	5	5	6	6	7	4	1	5	1	2	2	5	5	3	2	3	4	2	2	4	6	4	4	4	4	2	4
48	31	5941582	654	12	1	1	1	5	5	4	2	6	3	1	1	6	17	7	7	7	7	7	7	5	5	4	7	6	7	7	7	7	7	5	7	2	1	1	1	2	2	7	1	1	1	1	1	1	1	1	1	1	4	2	1	7
49	31	5941582	358	12	1	1	1	6	1	4	1	3	3	2	3	45		7	6	5	6	6	6	7	6	6	6	6	6	6	6	5	6	6	6	1	1	1	1	1	1	7	2	2	2	2	1	1	1	1	2	2	4	3	3	2
41	31	5941582	8	12	1	1	1	5	5	6	1	6	1	1	3	36		7	7	7	6	7	6	6	6	6				7	7	6	4	5	6	1	1	1	1	1	2	7	3	4	3	3	1	1	1	1	2	1	4	4	2	
411	31	5941582	1742	12	5	1	1	6	1	4	5	6	4	2	3	44	8	7	6	7	7	6	6	7	6	6	6	6	5	7	6	7	7	7	7	1	2	1	2	2	2	7	3	4	5	6	5	6	4	6	5	2	6	7	4	3
